# Distinct and Cooperative Activities of HESO1 and URT1 Nucleotidyl Transferases in MicroRNA Turnover in *Arabidopsis*

**DOI:** 10.1371/journal.pgen.1005119

**Published:** 2015-04-30

**Authors:** Bin Tu, Li Liu, Chi Xu, Jixian Zhai, Shengben Li, Miguel A. Lopez, Yuanyuan Zhao, Yu Yu, Vanitharani Ramachandran, Guodong Ren, Bin Yu, Shigui Li, Blake C. Meyers, Beixin Mo, Xuemei Chen

**Affiliations:** 1 Department of Botany and Plant Sciences, Institute of Integrative Genome Biology, University of California, Riverside, Riverside, California, United States of America; 2 Rice Research Institute, Sichuan Agricultural University, Chengdu Wenjiang, Sichuan, China; 3 Shenzhen Key Laboratory of Microbial Genetic Engineering, College of Life Sciences, Shenzhen University, Shenzhen, China; 4 Department of Plant & Soil Sciences, and Delaware Biotechnology Institute, University of Delaware, Newark, Delaware, United States of America; 5 Center for Plant Science Innovation & School of Biological Sciences, University of Nebraska-Lincoln, Lincoln, Nebraska, United States of America; 6 Howard Hughes Medical Institute, University of California, Riverside, Riverside, California, United States of America; Pennsylvania State University UNITED STATES

## Abstract

3’ uridylation is increasingly recognized as a conserved RNA modification process associated with RNA turnover in eukaryotes. 2’-*O*-methylation on the 3’ terminal ribose protects micro(mi)RNAs from 3’ truncation and 3’ uridylation in *Arabidopsis*. Previously, we identified HESO1 as the nucleotidyl transferase that uridylates most unmethylated miRNAs *in vivo*, but substantial 3’ tailing of miRNAs still remains in *heso1* loss-of-function mutants. In this study, we found that among nine other potential nucleotidyl transferases, UTP:RNA URIDYLYLTRANSFERASE 1 (URT1) is the single most predominant nucleotidyl transferase that tails miRNAs. URT1 and HESO1 prefer substrates with different 3’ end nucleotides *in vitro* and act cooperatively to tail different forms of the same miRNAs *in vivo*. Moreover, both HESO1 and URT1 exhibit nucleotidyl transferase activity on AGO1-bound miRNAs. Although these enzymes are able to add long tails to AGO1-bound miRNAs, the tailed miRNAs remain associated with AGO1. Moreover, tailing of AGO1-bound miRNA165/6 drastically reduces the slicing activity of AGO1-miR165/6, suggesting that tailing reduces miRNA activity. However, monouridylation of miR171a by URT1 endows the miRNA the ability to trigger the biogenesis of secondary siRNAs. Therefore, 3’ tailing could affect the activities of miRNAs in addition to leading to miRNA degradation.

## Author Summary

The tailing of RNAs with non-templated uridines, known as uridylation, is often associated with RNA degradation. We previously identified HESO1 as a nucleotidyl transferase that uridylates microRNAs (miRNAs) to lead to their degradation in Arabidopsis. But HESO1 cannot account for all the miRNA uridylation activity *in vivo*. Here, we have uncovered UTP:RNA URIDYLYLTRANSFERASE 1 (URT1) as another nucleotidyl transferase that uridylates miRNAs. HESO1 and URT1 have different substrate preferences and act cooperatively to tail miRNAs. We show that both enzymes are able to act on ARGONAUTE1 (AGO1)-bound miRNAs and that the tailed miRNAs stay bound by AGO1. We show that URT1-mediated tailing affects the activities of miR165/6 and miR171a differently. This study reveals intricate miRNA uridylation processes as well as functional outcomes of miRNA uridylation.

## Introduction

The three major types of small RNAs in eukaryotes, microRNAs (miRNAs), small interfering RNAs (siRNAs) and piwi-interacting RNAs (piRNAs), impact many biological processes such as development, self/non-self recognition, genome stability, and adaption to environment. Given the widespread and indispensible functions of small RNAs, it is crucial to understand their biogenesis and turnover. A common step in the biogenesis of miRNAs and siRNAs in plants, as well as piRNAs and certain endogenous siRNAs in animals, is 2’-*O*-methylation on the 3’ terminal ribose by the small RNA methyltransferase HUA ENHANCER1 (HEN1) [1,2,3,4,5,6,7]. In *Arabidopsis hen1* mutants, miRNAs and siRNAs become 3’ truncated, 3’ uridylated, and reduced in abundance [8]. Similarly, in animal *hen1* mutants, piRNAs and/or siRNAs become 3’ truncated and 3’ uridylated [2,3,4,6]. This indicates that 2’-*O*-methylation protects small RNAs from 3’ truncation by an exonuclease(s) and 3’ tailing by a nucleotidyl transferase(s).

In a previous study, we identified *Arabidopsis* HEN1 SUPPRESSOR1 (HESO1) as a nucleotidyl transferase responsible for miRNA uridylation *in vivo* [9,10]. Loss of function in *HESO1* results in reduced 3’ tailing and increased abundance for most miRNAs in *hen1* backgrounds [9,10], indicating that tailing leads to miRNA degradation. Small RNA profiling in *hen1-8*, a partial loss-of-function *hen1* mutant [11], and the *hen1-8 heso1-1* double mutant reveals that 3’ truncation happens independently of 3’ uridylation, and that 3’ uridylation occurs on both full-length and 3’ truncated species [10]. Among the ten genes encoding potential nucleotidyl transferases, *HESO1* is the only one whose loss of function partially suppresses the morphological defects of *hen1* mutants [9,10,12], suggesting that HESO1 is the major miRNA uridylation enzyme. However, despite *heso1-1* being a null allele [10], considerable levels of miRNA uridylation were detected by small RNA profiling in the *hen1-8 heso1-1* double mutant [10], indicating that (an)other nucleotidyl transferases could tail unmethylated miRNAs.

*In vivo*, a miRNA exists in miRISC (miRNA-induced silencing complex) in which the miRNA is bound by an ARGONAUTE (AGO) protein. In *Arabidopsis*, AGO1 is the effector for almost all miRNAs [13,14]. Molecular genetic evidence suggests that uridylation occurs on AGO1-bound miRNAs *in vivo*. First, 3’ truncated and tailed miRNAs in *hen1* mutants are bound by AGO1 *in vivo* [12,15]. Second, a partial loss-of-function *ago1* mutation, *ago1-11*, suppresses the 3’ truncation and tailing of miRNAs in the weak *hen1-2* mutant [15]. Moreover, HESO1 was shown to uridylate an *in vitro* reconstituted miRISC [16].

Given that uridylated miRNAs are associated with AGO1, uridylation may also alter the activities of miRNAs in addition to its role in miRNA degradation. Some *Arabidopsis* miRNAs efficiently trigger the production of phased secondary siRNAs (phasiRNAs) from their target transcripts, and some of the phasiRNAs can also regulate genes *in trans* and are named *trans*-acting siRNAs (ta-siRNAs) [17,18,19]. A common feature of the trigger miRNAs—their 22 nt length—endows them with the ability to generate phasiRNAs [20,21]. For example, miR173 is predominantly 22 nt *in vivo* and initiates ta-siRNA biogenesis from two noncoding transcripts *TAS1* and *TAS2* [17,18,19]. In a recent study [15], it was found that miR171a becomes predominantly 22 nt in *hen1* mutants and initiates the biogenesis of phasiRNAs from its targets At2g45160 and At3g60630, suggesting that mono-uridylation of miR171a allows it to trigger phasiRNA biogenesis. But the enzyme that mono-uridylates miR171a in *hen1* mutants is not HESO1 and remains unknown [15]. In *hen1* mutants, despite various degrees of tailing of many miRNAs, only miR171 family members acquire the ability to trigger phasiRNA biogenesis [15]. The effects of tailing on the activities of other miRNAs remain unknown.

Most of the nine *HESO1* paralogs, which we refer to as *NUCLEOTIDYL TRANSFERASE PROTEIN* (*NTP*) genes unless they have been previously named otherwise (S1 Fig), remain uncharacterized. One gene (At2g45620) was shown to encode a nucleotidyl transferase and named *UTP*:*RNA URIDYLYLTRANSFERASE 1* (*URT1*) [22]. URT1 was found to uridylate messenger RNAs that have an oligoadenylate tail [22].

In this study, we identified URT1 as the nucleotidyl transferase with the second highest impact on miRNA uridylation among ten nucleotidyl transferases in *Arabidopsis*. URT1 and HESO1 prefer miRNA substrates with different 3’ nucleotides *in vitro*, and act on different size variants of the same miRNAs *in vivo*. We found that URT1 and HESO1 act sequentially on some miRNAs, with URT1 mono-uridylating the miRNAs followed by their further uridylation by HESO1. Both HESO1 and URT1 are able to tail AGO1-bound miRNAs and the uridylated species stay associated with AGO1. The tailing of AGO1-bound miR165/6 reduces its slicing activity while the monouridylation of miR171a endows an ability to trigger the biogenesis of phasiRNAs. Thus, miRNA tailing affects the activities of miRNAs in addition to causing miRNA degradation.

## Results

### *URT1* is responsible for the uridylation of some miRNAs in *hen1-8*

The presence of substantial levels of miRNA uridylation in *hen1-8 heso1-1* implied that (an)other nucleotidyl transferases can also tail miRNAs. To identify the nucleotidyl transferase(s), we sought to determine the effects of mutations in each of the nine *NTP* genes (S1 Table and S1A Fig) on miRNA uridylation. A mutation in each of the nine *NTP* genes was combined with *hen1-8* and small RNA libraries were constructed from the nine double mutants as well as *hen1-8* (S2 Table). Levels of tailing were calculated as the ratio of the number of reads with tails over the number of total reads for a particular miRNA.

**Fig 1:**
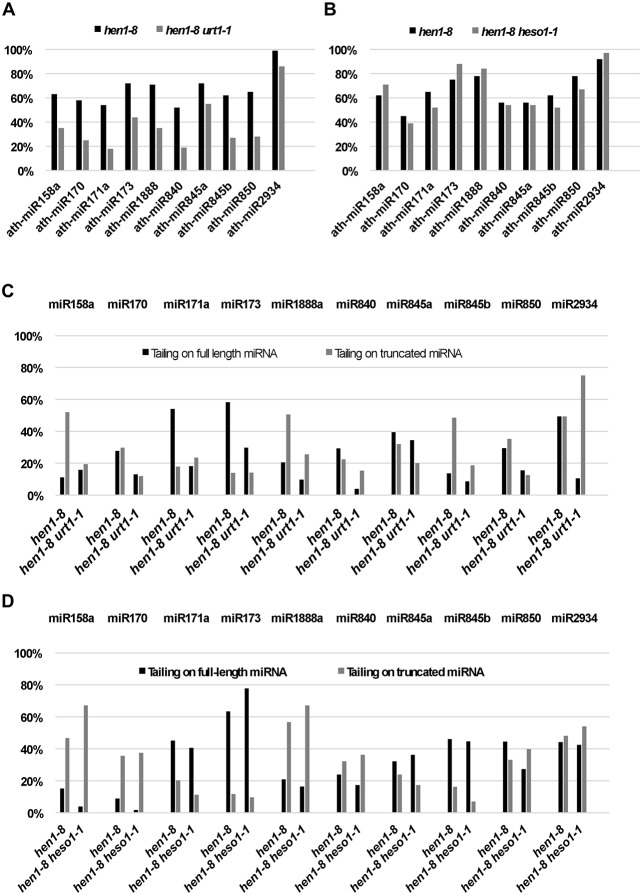
Quantification of miRNA tailing by small RNA high throughput sequencing. (A) The proportions of tailed reads for ten miRNAs in *hen1-8* and *hen1-8 urt1-1*. (B) The proportions of tailed reads for the same ten miRNAs in *hen1-8* and *hen1-8 heso1-1*. The proportions of tailed species were calculated as %(sum of tailed-only and truncated-and-tailed reads divided by total read number). (C-D) The levels of tailing on full-length and 3’ truncated species from ten miRNAs in *hen1-8* and *hen1-8 urt1-1* (C), and *hen1-8* and *hen1-8 heso1-1* (D). The levels of tailing on full-length miRNAs were calculated as a percentage (number of tailed-only reads divided by sum of tailed and non-tailed full-length reads). The levels of tailing on truncated miRNAs were calculated as a percentage (number of truncated-and-tailed reads divided by sum of truncated-and-tailed and truncated-only reads).

The mutation in *URT1*, *urt1-1*, was found to reduce the tailing of a few miRNAs. Among 107 miRNAs that were detected at 30 reads per million or greater abundance in both *hen1-8* and *hen1-8 urt1-1* libraries, ten showed reduced tailing in *hen1-8 urt1-1* (Fig 1A). The effects of *urt1-1* on the tailing of a small number of miRNAs contrasted the widespread effects of *heso1* mutations [9,10]. This suggested that HESO1 could act on most miRNAs in *hen1-8 urt1-1* except for a few miRNAs. Indeed, for the miRNAs showing reduced tailing in *hen1-8 urt1-1*, the *heso1-1* mutation had no or little effect on them whereas it had a strong effect on other miRNAs (Fig 1B; [10]). When we separately quantified tailing on full-length and 3’ truncated species, we found that *urt1-1* and *heso1-1* had distinct effects on the different forms of the same miRNAs (Fig 1C and 1D). For example, *urt1-1* strongly reduced 3’ tailing of full-length miR171a and 3’ truncated miR158a, but *heso1-1* affected the tailing of 3’ truncated miR171a and full-length miR158a (Fig 1C and 1D). Therefore, the two proteins appeared to prefer different miRNAs or different forms of the same miRNAs *in vivo*. Unlike *heso1-1*, which partially rescues the *hen1-8* morphological phenotype [10], *urt1-1* does not rescue the *hen1-8* morphological phenotype (S2 Fig).

**Fig 2:**
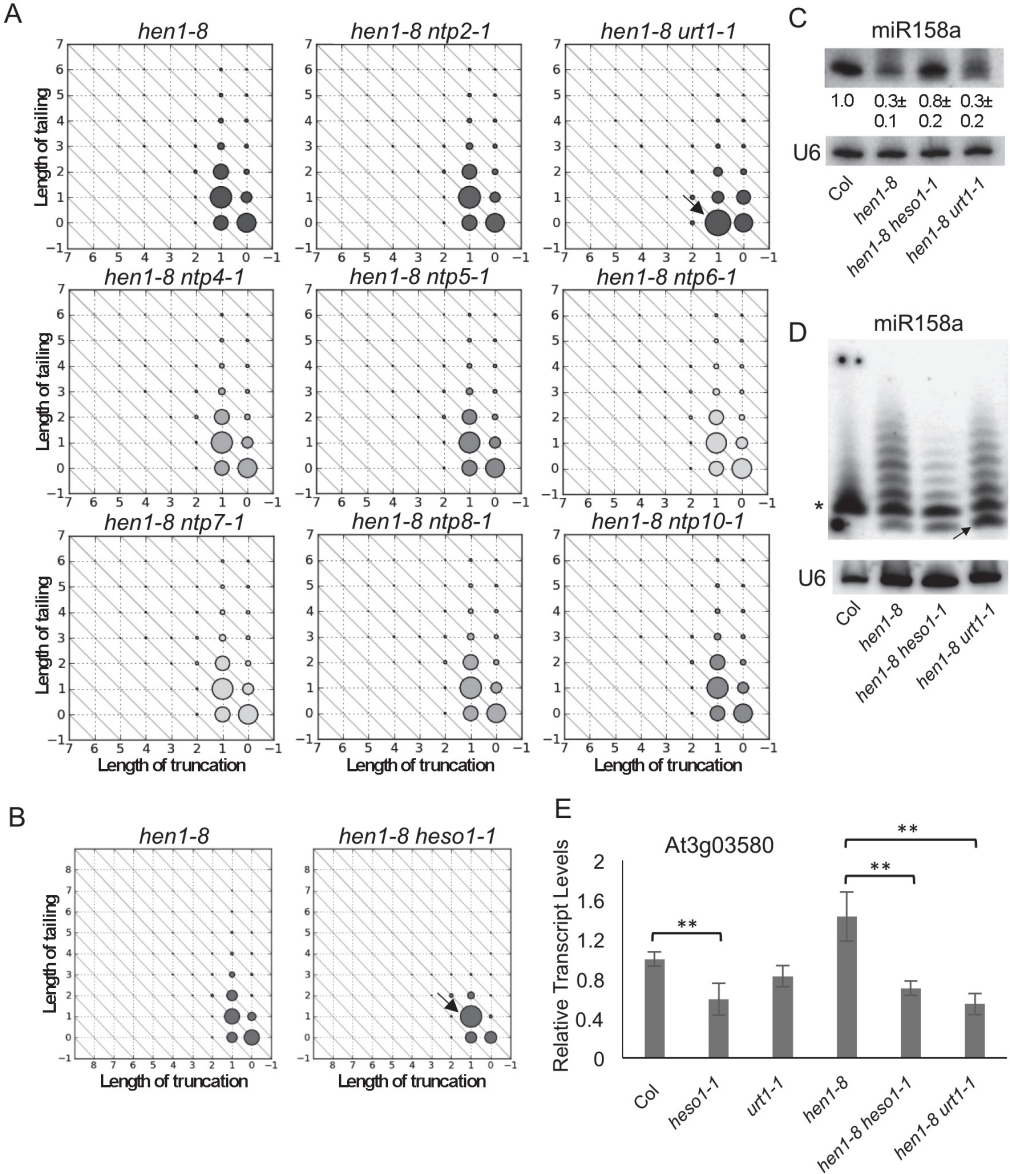
Distinct and coordinated tailing of miR158 by URT1 and HESO1. (A-B) Diagrams that represent the status of 3’ truncation and 3’ tailing of miR158a in various genotypes *in vivo* as determined by small RNA sequencing. In the diagrams, the X and Y axes represent the number of nucleotides truncated from, and tailed onto, the miRNA 3’ end, respectively. The sizes of the circles indicate the relative abundance of the miR158 variants. Nine and two genotypes were examined in (A) and (B), respectively, as indicated. In each figure, samples from the different genotypes were processed at the same time for small RNA library construction. Altogether in (A) and (B), mutations in nine *NUCLEOTIDYL TRANSFERASE PROTEIN* (*NTP*) genes, including *URT1* and *HESO1*, were examined. Refer to S3 Fig for results on *hen1-8 mee44*. A miR158 species with a 1-nt truncation (marked by an arrow in (A)) accumulates in *hen1-8 urt1-1*. The miR158 species at the (1,1) position (marked by an arrow in (B)) accumulates in *hen1-8 heso1-1*. (C-D) The accumulation of miR158 in *hen1-8*, *hen1-8 heso1-1*, *hen1-8 urt1-1* as determined by northern blotting. Total RNAs (5 μg for Col and 50 μg for all *hen1* genotypes) were resolved in a short (C) and a long (D) polyacrylamide gel, the latter allowing the separation of miR158 species that differ by 1 nt. U6 serves as a loading control. In (C), the relative abundance of miR158, shown as mean +/- SD below the miR158 gel image, was calculated from three biological replicates. In (D), the arrow marks the 1-nt truncated species, whereas the asterisk indicates the 20-nt band. This band is deduced to represent 20-nt RNAs as it is the size of miR158 in wild type (Col), and miR158 is known to be 20-nt long in wild type. (E) Relative transcript levels of At3g03580, a target of miR158, as determined by real-time RT-PCR. *ACTIN1* was used as an internal control. Error bars representing SD were calculated from three biological replicates. Significant differences are indicated by ** (P value < 0.01) for the pairs of genotypes that were compared.

In contrast to mutations in *HESO1* or *URT1*, mutations in the other eight *NTP* genes did not cause any discernable effects on miRNA tailing (S3 Fig). As an example, miR158 showed reduced tailing in *hen1-8 urt1-1* as compared to *hen1-8* (arrow in Fig 2A). Patterns of miR158 3’ truncation and tailing in the other eight *hen1-8 ntp* double mutants were identical to each other and to *hen1-8* (Fig 2A and S4A Fig). The nearly identical patterns of *hen1-8* and seven *hen1-8 ntp* genotypes in Fig 2A also reflected the reproducibility of the high throughput sequencing-based quantification of miRNA 3’ truncation and tailing.

### URT1 exhibits nucleotidyl transferase activity on unmethylated miRNA *in vitro*

**Fig 3:**
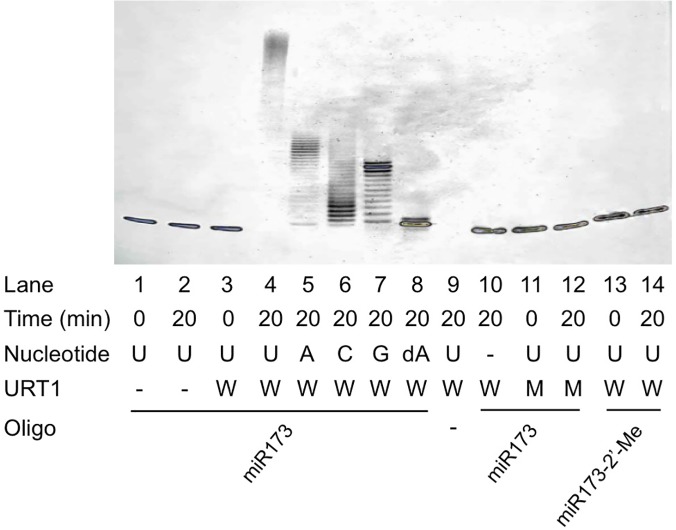
Nucleotidyl transferase assays for URT1. Recombinant His-tagged wild type URT1 or a catalytic mutant was included in the reactions. The wild type and mutant proteins are indicated by “W” and “M”, respectively. The proteins were incubated with an RNA oligonucleotide (oligo) in the presence of a nucleotide triphosphate, and the products were resolved on 15% denaturing polyacrylamide gels. miR173 and miR173-2’-Me are un-methylated and methylated, respectively, on the 2’ OH of the 3’ terminal ribose. Nucleotidyl transferase activity is represented by the presence of higher molecular weight bands relative to the input miRNA. Note that in lane 4, the substrate RNA was completely converted to higher molecular weight products at the top of the gel. The ‘‘-” signs indicate the absence of the nucleotide triphosphate, protein, or RNA oligonucleotide.

URT1 was previously shown to have RNA nucleotidyl transferase activity *in vitro* and uridylate oligoadenylated RNAs *in vivo* [22]. To confirm this activity and to examine its substrate preferences, we expressed and purified recombinant, 6XHis-tagged URT1 from *E*. *coli* (S1C Fig). The recombinant protein was incubated with synthetic, unmethylated miR173 in the presence of different ribonucleotide triphosphates. URT1 was able to add multiple ribonucleotides to miR173 (Fig 3, lanes 4–7), confirming that URT1 is a nucleotidyl transferase. The lengths of the products were longest when UTP was in the reaction (Fig 3, lanes 4–7), indicating that URT1, like HESO1 [9,10], prefers UTP. To rule out the possibility that the nucleotidyl transferase activity was due to a contaminating protein from *E*. *coli*, two conserved aspartate residues in the nucleotidyl transferase domain were mutated to alanine (S1B Fig). The mutant protein, 6XHis-URT1m was expressed in *E*. *coli* and purified as was the wild-type protein (S1C Fig). His-URT1m failed to tail miR173 (Fig 3, lanes 11–12).

We next investigated URT1’s requirement for the 2’ OH of the 3’ terminal ribose in the substrate miRNA. When deoxyadenosine triphosphate (dATP) was included as the only nucleotide in the reaction, URT1 added a single deoxyadenylate to miR173, but further nucleotide addition was inefficient (Fig 3, lane 8). This indicated that the 2’ OH on the 3’ terminal ribose is a feature of the substrate recognized by URT1. When 2’-*O*-methylated miR173 was incubated with URT1 in the presence of UTP, no tailing was observed (Fig 3, lanes 13–14). This indicated that URT1 activity, like that of HESO1 [10], is completely inhibited by 2’-*O*-methylation on its substrate RNA.

### URT1 and HESO1 act cooperatively on miR158 by tailing different forms

Small RNA profiling in *hen1-8 urt1-1* and *hen1-8 heso1-1* revealed that URT1 and HESO1 preferred different forms of miR158 (Fig 2A and 2B). Note that the various miR158 species will be referred to by their X and Y coordinates as shown in Fig 2A and 2B. In *hen1-8*, miR158 existed in full-length and 1-nt truncated forms (miR158 (0,0) and miR158 (1,0), respectively) and both underwent various degrees of tailing (Fig 2A). In *hen1-8 heso1-1*, tailing of the full-length form was greatly reduced, indicating that HESO1 was responsible for the tailing of full-length miR158 (Fig 2B). In *hen1-8 urt1-1*, full-length miR158 species with tails were unaffected, indicating that URT1 did not tail full-length miR158 (Fig 2A). In *hen1-8 heso1-1*, there was an increase in miR158 (1,1), a miR158 species with 1-nt truncation and a 1-nt tail (Fig 2B; arrow). Correspondingly, there was a decrease in the abundance of miR158 (1,1+), i.e., species of miR158 (1,1) with longer tails (Fig 2B). Therefore, HESO1 was responsible for tailing miR158 (1,1) but not miR158 (1,0). On the other hand, the abundance of miR158 (1,0) was increased with a corresponding decrease in miR158 (1,0+) species in *hen1-8 urt1-1* (Fig 2A). Taken together, these results suggested that 1) HESO1 but not URT1 tails full-length miR158; and 2) URT1 adds a 1-nt tail to the 1-nt truncated miR158 to result in miR158 (1,1), which is further tailed by HESO1.

To verify these results, northern blots were carried out on wild type, *hen1-8*, *hen1-8 urt1-1*, and *hen1-8 heso1-1*. When RNAs were separated with a 1-nt resolution, it was clear that the 20-nt miR158 band was the predominant band in *hen1-8 heso1-1* (Fig 2D). This 20-nt band corresponded to both miR158 (0,0) and miR158 (1,1) (Fig 2B), and the northern blot results were consistent with the small RNA profiling data. On the other hand, in *hen1-8 urt1-1*, the 1-nt truncated form of miR158 accumulated to a higher level relative to *hen1-8* (Fig 2D; arrow), consistent with the small RNA profiling data (Fig 2A; arrow). We also quantified the levels of miR158 in the four genotypes by not resolving the miR158 size variants in northern blots. Consistent with previous results, the amount of miR158 was higher in *hen1-8 heso1-1* as compared to *hen1-8* (Fig 2C). *urt1-1* did not lead to an increase in miR158 abundance (Fig 2C).

miR158 is only partially methylated in wild type and is subjected to uridylation by both HESO1 and URT1 even in wild type background ([15]; S4B Fig). As in the *hen1-8* background, HESO1 and URT1 act on full-length and 1-nt truncated miR158, respectively, in wild type (S4B Fig).

Next, we tested whether URT1 or HESO1 impacts the activity of miR158 by examining the expression of the miR158 target gene At3g03580. The *heso1-1* mutation caused a reduction in At3g03580 transcript levels as compared to wild type, but the *urt1-1* mutation did not have a significant effect (Fig 2E). The levels of the At3g03580 transcript were reduced in *hen1-8 heso1-1* relative to *hen1-8* (Fig 2E), consistent with higher miR158 levels in this genotype (Fig 2C). Interestingly, the expression of At3g03580 was decreased also in *hen1-8 urt1-1* (Fig 2E), despite the fact that miR158 levels were similar between *hen1-8 urt1-1* and *hen1-8* (Fig 2C). This prompted us to test whether tailing affects the slicer activity of miRNAs (see below).

### URT1 and HESO1 sequentially tail miR173 and reduce the biogenesis of ta-siRNAs

**Fig 4:**
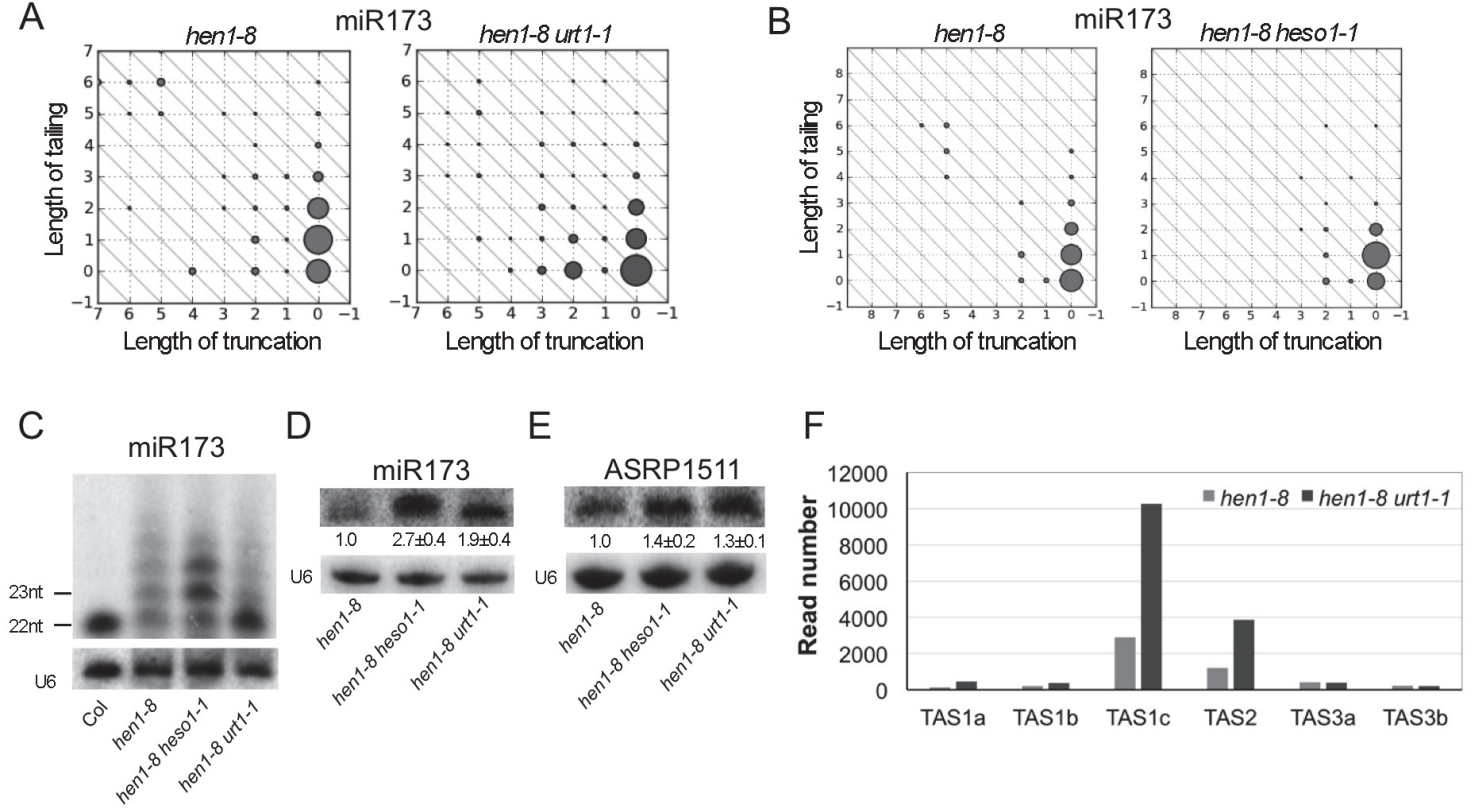
Sequential tailing of miR173 in *hen1* by URT1 and HESO1 and its impacts on ta-siRNA biogenesis. (A-B) The accumulation of various miR173 species as determined by small RNA sequencing. Refer to the Fig 3 legend for the description of the diagrams. The two samples in (A) or (B) were processed in the same experiment, but (A) and (B) were from two different experiments. In *hen1-8 urt1-1*, the full-length miR173 species at position (0,0) accumulates to higher levels in comparison to *hen1-8*. In *hen1-8 heso1-1*, the miR173 species with a 1-nt tail at position (0,1) accumulates to higher levels as compared to *hen1-8*. (C) The accumulation of miR173 variants of different sizes in various genotypes was determined by northern blotting. 5 μg and 50 μg of total RNAs were used for Col (wild type) and *hen1-8* genotypes, respectively. (D) The amount of miR173 in *hen1-8*, *hen1-8 heso1-1* and *hen1-8 urt1-1* was determined by northern blotting. RNAs were resolved in a short gel so as not to separate the size variants. The numbers below the miR173 gel image represent the relative abundance of miR173 in various genotypes. The error bars represent SD and were calculated from three biological replicates. (E) The accumulation of ASRP1511, a ta-siRNA from the *TAS2* locus, as determined by northern blotting. The error bars represent SD and were calculated from three biological replicates. (F) The abundance of ta-siRNAs from various *TAS* loci in *hen1-8* and *hen1-8 urt1-1* as determined by small RNA sequencing. The Y axis represents relative read number of the ta-siRNAs in the two genotypes normalized to total small RNAs.

Small RNA sequencing revealed that URT1 and HESO1 acted sequentially to tail miR173. miR173 is largely non-tailed in wild type (S4C Fig). In *hen1-8*, full-length, 22-nt miR173 was tailed to various larger sizes (Fig 4A and 4B). Both *heso1-1* and *urt1-1* affected miR173 tailing but in different ways. In *hen1-8 heso1-1*, the mono-uridylated, 23-nt form of miR173 accumulated predominantly (Fig 4B), indicating that HESO1 preferentially used this 23-nt form as the substrate for uridylation *in vivo*. The enzyme that performed the mono-uridylation of miR173 to produce the 23-nt form was URT1, as the 22-nt form of miR173 was the predominant species in *hen1-8 urt1-1* (Fig 4A). These observations from small RNA sequencing were confirmed by northern blotting. The 23-nt and larger species were predominant in *hen1-8 heso1-1*, whereas the 22-nt species was predominant in *hen1-8 urt1-1* (Fig 4C). Taken together, these results indicate that URT1 performs mono-uridylation of miR173; subsequently, HESO1 preferentially uridylates the 23-nt miR173. Moreover, consistent with uridylation leading to miRNA degradation, miR173 levels were increased in both *hen1-8 heso1-1* and *hen1-8 urt1-1* relative to *hen1-8* (Fig 4D). When a *URT1-GFP* transgene driven by the *URT1* promoter was introduced into *hen1-8 urt1-1*, the increase in miR173 levels was rescued in two independent transgenic lines (S5A Fig). In a *HEN1* background, neither *heso1-1* nor *urt1-1* affected the levels of miR173 (S5B Fig).

As a 22-nt miRNA, miR173 triggers the biogenesis of ta-siRNAs from *TAS1* and *TAS2* loci [17,18,19,20,21]. We next examined whether *URT1* impacted ta-siRNAs whose biogenesis requires miR173. Northern blots were performed to detect ASRP1511, a ta-siRNA from the *TAS2* locus [17]. ASRP1511 levels were higher in *hen1-8 urt1-1* or *hen1-8 heso1-1* than in *hen1-8* (Fig 4E). The increase in *TAS2* ta-siRNA levels caused by *urt1-1* and *heso1-1* mutations could be multifactorial—an increase in miR173 levels in both *hen1-8 urt1-1* and *hen1-8 heso1-1* (Fig 4D), an increase in the 22-nt form of miR173 in *hen1-8 urt1-1* (Fig 4C), and compromised uridylation of the ta-siRNAs themselves in *hen1-8 heso1-1* [10].

We also examined the abundance of *TAS1*, *TAS2* and *TAS3* ta-siRNAs in small RNA libraries. *TAS3* ta-siRNAs were included as a control, as their trigger, miR390 [23], was not affected by the *urt1-1* mutation in terms of tailing (S3 Fig). When normalized to total small RNAs, the abundance of *TAS1* and *TAS2* ta-siRNAs was much higher in *hen1-8 urt1-1* than in *hen1-8*, while that of *TAS3* ta-siRNAs was not affected (Fig 4F). *hen1-8 heso1-1* was not included in the analysis as the global increase in miRNA and ta-siRNA levels prevented effective normalization.

### URT1 and HESO1 have different substrate specificities *in vitro*

The above small RNA sequencing results described above revealed differential preferences for miR158 and miR173 forms by HESO1 and URT1. For example, *in vivo*, full-length miR158 is mainly tailed by HESO1 while 1-nt truncated miR158 (miR158-1) is mainly tailed by URT1. To determine whether this reflected different substrate preferences for the two enzymes, we examined HESO1 and URT1 activities on miR158 and miR158-1 *in vitro*. As miR158-1 differs from miR158 both in length and in the nature of the 3’ nucleotide (miR158-1 ends in C while miR158 ends in A), we also included three full-length miR158 forms ending in C, G, or U (referred to as miR158A-C, miR158A-G, and miR158A-U, respectively) in comparison to the natural miR158 ending in A. As the two enzymes required different buffer conditions for optimal activities, it was not reasonable to compare the activities of the two enzymes on the same substrate. But it was possible to compare the activities of an enzyme on different substrates. When comparing nucleotidyl transferase activities on different substrates, the disappearance of the substrate over a time course was considered as the criteria for preference for the substrate.

**Fig 5:**
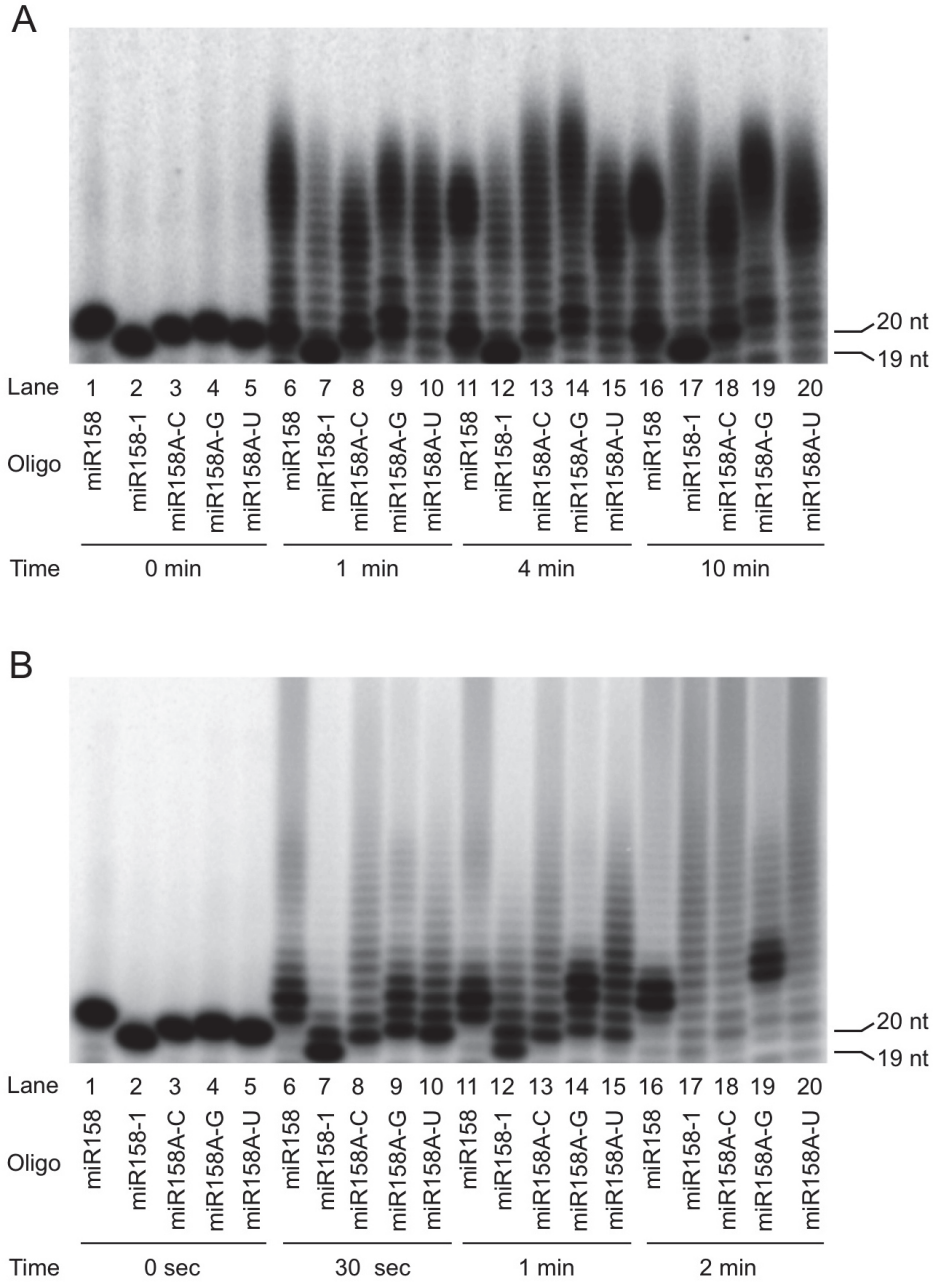
Nucleotidyl transferase assays to evaluate the substrate preferences by HESO1 and URT1. Reactions were conducted with HESO1 (A) or URT1 (B) and five RNA oligonucleotides (oligo) in a time course as indicated. The sizes of the input miRNAs are indicated (19 nt for miR158-1 and 20 nt for the full-length forms of miR158). The RNAs were 5’ ^32^P-labeled and gel purified prior to the reactions. The substrate/enzyme ratio was 1:2 for HESO1 and 1:8 for URT1. The sizes of the RNA substrates are indicated.

Among the four full-length miR158 forms (Fig 5A, lanes 1, 3–4), HESO1 had a clear preference for miR158A-U, as reflected by the disappearance of this substrate but not others at the earliest time point (Fig 5A, lanes 6, 9–10). With longer time points, it was apparent that miR158A-G was the second most preferred substrate. At 4 min, less non-tailed miR158A-G was present compared to non-tailed miR158 or miR158A-C (Fig 5A, compare lane 14 to lane 11 or 13). At 10 min, while substantial amount of non-tailed miR158 or miR158A-C was present, almost all miR158A-G was tailed (Fig 5A, compare lane 19 to lane 16 or 18).

URT1 showed a strong preference for miR158 ending in A, as the substrate was depleted at the earliest time point while the other miR158 forms still remained (Fig 5B, compare lane 6 to lanes 8–10). The preference for A-ending substrates is consistent with the finding that URT1 uridylates mRNAs with oligoadenylate tails *in vivo* [22]. The other three forms of miR158 (miR158A-C, miR158A-G, and miR158A-U) were similarly used by URT1 in the reaction time course (Fig 5B, lanes 8–10, 13–15, and 18–20).

miR158-1 was the least favored substrate among the five miR158 forms tested for both HESO1 and URT1 (Fig 5A and 5B). But miR158-1 was better tolerated by URT1 than by HESO1, as it was tailed only slightly more slowly than miR158A-C, miR158A-G, or miR158A-U by URT1 (Fig 5B, compare lane 12 to lanes 13–14, and lane 17 to lanes 18–20). But it was barely tailed by HESO1 when the full-length miR158 forms were substantially tailed (Fig 5A, compare lane 12 to lanes 13–14, and lane 17 to lanes 18–20). This difference of the two enzymes could potentially explain the *in vivo* tailing of miR158-1 by URT1 but not HESO1.

We next tested whether tailing of miR158 or miR158-1 was more efficient with both HESO1 and URT1 acting together. In order to compare the activities of different enzymes, we had to use a single buffer, and we chose the URT1 buffer for all three reactions, URT1 alone, HESO1 alone, and URT1 and HESO1 together. Indeed, for both miR158 and miR158-1, the two enzymes together tailed the miRNAs better than each enzyme alone (S6 Fig).

### URT1 and HESO1 act on AGO1-bound miRNAs

**Fig 6:**
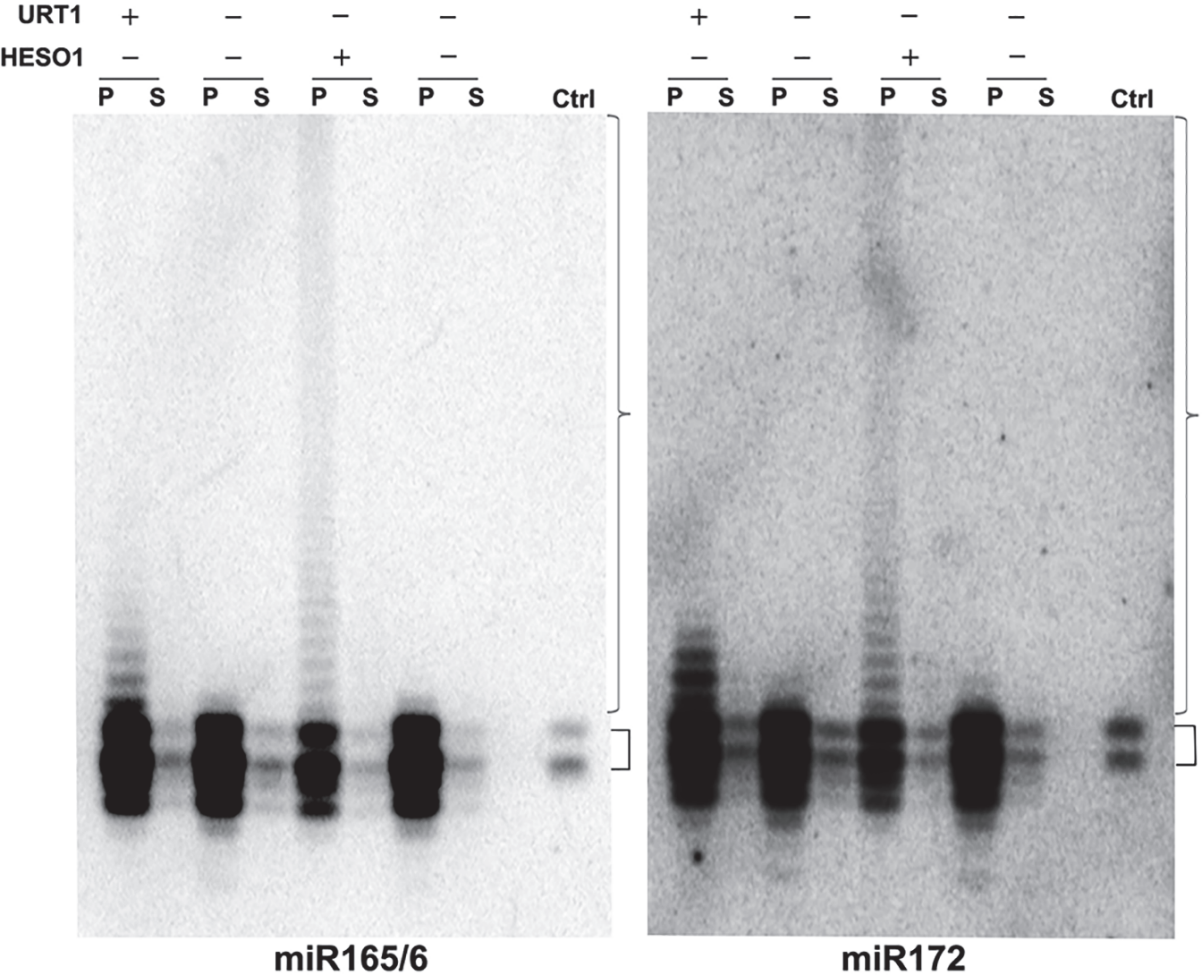
URT1 and HESO1 can tail AGO1-bound miRNAs. Nucleotidyl transferase assays were conducted with recombinant URT1, HESO1, or no enzyme as indicated, AGO1 immunoprecipitate (IP) as the substrate, and cold UTP. AGO1 IP was prepared from *hen1-2 heso1-2 urt1-3* so that the associated miRNAs lack 2’-*O*-methylation or tailing. After the reactions, the reaction mixes were separated into the precipitate (P) and supernatant (S) fractions and the RNAs were isolated separately from the two fractions and subjected to northern blotting to detect miR165/6 and miR172. The lanes “ctrl” were total RNA from wild type included during gel electrophoresis. The smaller brackets indicate full-length miRNAs from wild type. Both full-length and 3’ truncated miRNAs were present in the AGO1 IP from *hen1-2 heso1-2 urt1-3*. The larger brackets indicate tailed miRNAs.

We previously showed that HESO1 is able to tail miR166 in a reconstituted AGO1-miR166 RISC [16]. We sought to determine whether URT1 had the capability to act on AGO1-bound miRNAs and to confirm the ability of HESO1 to tail AGO1-bound miRNAs in native miRISCs. To obtain native, AGO1-bound, but unmethylated miRNAs for use as substrates, we took advantage of the fact that miRNAs lack methylation and show drastically reduced uridylation in the *hen1-2 heso1-2 urt1-3* background (see Wang *et al*., companion manuscript). AGO1 was immunoprecipated from this genetic background, and the immunoprecipate (IP) was subjected to western blotting to detect AGO1 and northern blotting to detect miR165/6 and miR172. Both AGO1 and the two miRNAs were found in the IP (Fig 6 and S7 Fig). In comparison to the miRNA species in wild-type plants, the miRNAs in the AGO1 IP from *hen1-2 heso1-2 urt1-3* included both full-length and truncated species (Fig 6).

The AGO1 IP was used as a substrate in reactions with URT1 or HESO1 and cold UTP. After the reactions, AGO1 was collected in the precipitate, and RNAs in both the precipitate (associated with AGO1) and the supernatant (released from AGO1) were detected by northern blotting using probes against miR165/6 or miR172. HESO1 was able to tail the two miRNAs in native miRISCs (Fig 6). In fact, the lengths of tails added by HESO1 exceeded 100 nucleotides. Despite such long tails, the tailed miRNAs were still bound by AGO1, as they were present in the precipitate rather than the supernatant (Fig 6). URT1 was also able to tail AGO1-bound miR165/6 and miR172 (Fig 6), but the lengths of tails introduced by URT1 were much shorter than those generated by HESO1. The miRNAs tailed by URT1 were also associated with AGO1 (Fig 6).

### URT1 tails miR171a to 22 nt in *hen1* to initiate phasiRNA biogenesis

**Fig 7:**
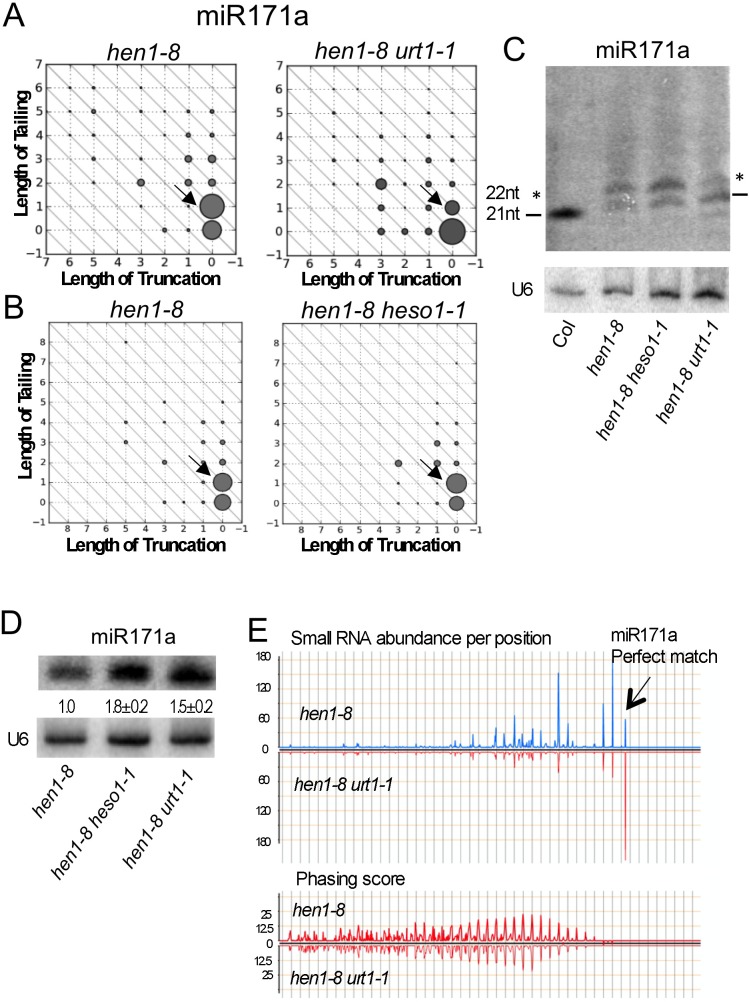
Tailing of miR171a in *hen1* by URT1 triggers phasiRNA biogenesis. (A-B) The accumulation of various miR171a species as determined by small RNA sequencing. Refer to the Fig 2 legend for the description of the diagrams. The two samples in (A) or (B) were processed in the same experiment, but (A) and (B) were from two different experiments. The 22-nt miR171a form is marked by an arrow in each of the diagrams. This form is reduced in *hen1-8 urt1-1* but not *hen1-8 heso1-1*. (C) The accumulation of miR171a variants of different sizes in various genotypes was determined by northern blotting. 5 μg and 50 μg of total RNAs were used for Col (wild type) and *hen1-8* genotypes, respectively. The 21-nt bands are marked by the two dashes; the 22-nt bands are marked by the two asterisks. (D) The amount of miR171a in *hen1-8*, *hen1-8 heso1-1* and *hen1-8 urt1-1* was determined by northern blotting. RNAs were resolved in a short gel so as not to separate the size variants. The numbers below the miR171a gel image represent the relative abundance of miR171a in various genotypes. The error bars represent SD and were calculated from three biological replicates. (E) The impact of the *urt1-1* mutation on phasiRNA production from the miR171a target gene At2g45160. The X axes in the two panels represent the transcript of At2g45160 in the 3’ to 5’ orientation from left to right. In the top panel, the relative abundance of secondary siRNAs from At2g45160 was plotted. The arrow marks the position of the miR171a-binding site in the transcript, which is 5’ to all the secondary siRNAs. In the bottom panel, the phasing scores of the secondary siRNAs were plotted, showing that the secondary siRNAs are phasiRNAs in both genotypes.

Given that both HESO1 and URT1 can tail miRNAs in native miRISCs and that tailed miRNAs in *hen1* mutants are associated with AGO1 *in vivo* [12,15], we asked whether tailed miRNAs could be functional. We previously observed that miR171a, which is 21-nt long (S4D Fig) and unable to trigger the biogenesis of secondary phasiRNAs from its target genes At2g45160 and At3g60630 in wild type, acquired this ability in *hen1* mutants [15]. According to small RNA sequencing, 22-nt and 21-nt forms of miR171a were the two most abundant forms in *hen1-1* and *hen1-8* mutants [15] (Fig 7A and 7B), and we hypothesized that the tailing of 21-nt miR171a to 22 nt endowed the ability to trigger phasiRNA biogenesis. But this hypothesis was not tested as the enzyme that tailed miR171a to 22 nt was unknown. It was not HESO1, as in *hen1-8 heso1-1*, the 22-nt form showed an increase in abundance, probably because further tailing of this form was reduced ([15]; Fig 7B). We found that this enzyme was URT1 since the 21-nt form became the most abundant form in *hen1-8 urt1-1* (Fig 7A). In fact, the over-accumulation of miR171a (0,0) and miR171a (0,1) in *hen1-8 urt1-1* and *hen1-8 heso1-1*, respectively (Fig 7A and 7B), indicates that URT1 tails full-length miR171a by one nucleotide *in vivo* and the 1-nt-tailed form is further tailed by HESO1.

To verify these results from small RNA sequencing, we performed northern blotting of miR171a at a single nucleotide resolution. In both *hen1-8* and *hen1-8 heso1-1*, the abundance of the 22-nt form was higher than that of the 21-nt form (Fig 7C). In *hen1-8 urt1-1*, the opposite was observed (Fig 7C). Therefore, URT1 was responsible for the production of 22-nt miR171a by tailing miR171a by one nucleotide. Moreover, consistent with the cooperative tailing of miR171a by both URT1 and HESO1, miR171a levels were higher in *hen1-8 urt1-1* and *hen1-8 heso1-1* as compared to *hen1-8* (Fig 7D).

Next, we examined whether the *urt1-1* mutation, which reduced the levels of 22-nt miR171a, affected the production of phasiRNAs triggered by miR171a in *hen1-8*. From *hen1-8* and *hen1-8 urt1-1* small RNA libraries, secondary siRNAs mapping to At2g45160 (a target of miR171a) were normalized to total small RNAs and plotted along the At2g45160 gene (Fig 7E, top panel). It was clear that these secondary siRNAs were reduced in abundance in *hen1-8 urt1-1*. When the secondary siRNAs were examined for their phasing status, i.e., whether they occurred in 21-nt intervals from one another, no difference was observed in the phasing scores in *hen1-8* and *hen1-8 urt1-1* (Fig 7E, bottom panel). This indicated that phasiRNAs were produced from At2g45160 in both genotypes, but their abundance was much lower in *hen1-8 urt1-1*. Therefore, the tailing of miR171a to 22 nt in *hen1* by URT1 promotes miR171a-triggered phasiRNA biogenesis.

### Tailing of miR165/6 by URT1 reduces its slicing activity

Many miRNAs are normally 21-nt in length and they are tailed to various sizes including 22 nt in *hen1* mutants. But miR171 is the only miRNA that acquires the ability to generate phasiRNAs in *hen1* mutants [15], suggesting that the 22-nt forms of most miRNAs are not functional. In addition, the expression of At3g03580, a target of miR158, was repressed more effectively in *hen1 urt1* than in *hen1* (Fig 2E), despite the fact that miR158 was similar in abundance in the two genotypes. This suggested that tailed forms of miR158 are not as effective in target repression.

**Fig 8:**
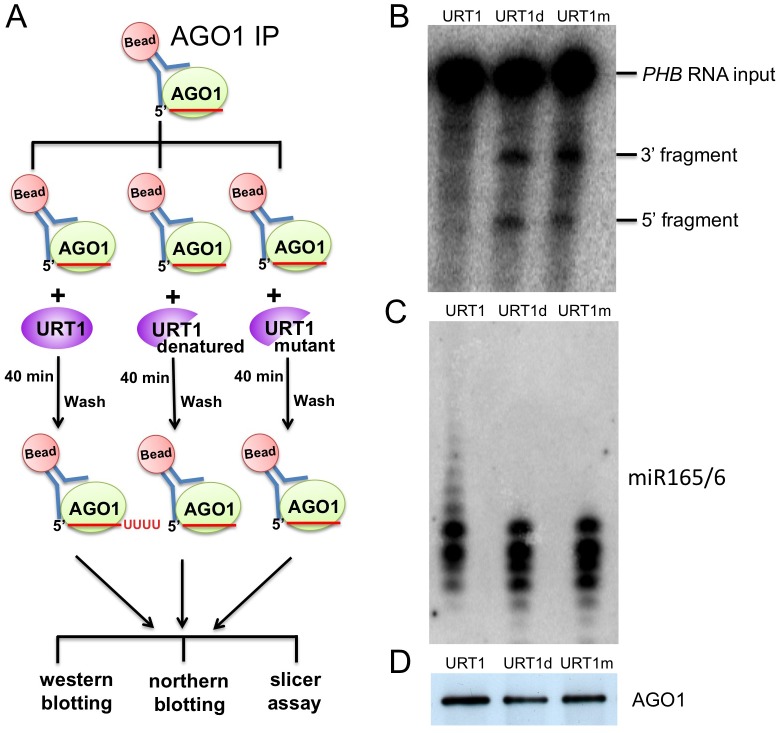
Tailing of AGO1-bound miR165/6 by URT1 drastically reduces its slicer activity. (A) An experimental scheme. AGO1 was immunoprecipitated from *hen1 heso1-2 urt1-3*. The IP was used as the substrate for tailing reactions by one of three enzymes. Each tailing reaction was then split into three equal portions for western blotting to quantify AGO1, northern blotting to determine the status of tailing of AGO1-bound miR165/6, and slicer assay. (B) Slicer assay following the tailing reactions. A portion of the *PHB* RNA containing the miR165/6 binding site was body-labeled with ^32^P and used as the input RNA. (C) Northern blotting to detect miR165/6 from the AGO1-bound fraction after the tailing reactions. (D) Western blotting to detect AGO1 after the tailing reactions.

We decided to examine the effects of tailing by URT1 on the activities of miRNAs *in vitro* using the slicer assay as the functional output of miRNAs. We immunoprecipitated AGO1 from *hen1-2 heso1-2 urt1-3*, and used the IP as the substrate for the tailing reaction with URT1. Following the tailing reaction, AGO1 was collected in the precipitate, washed, and incubated with a fragment of the *PHB* transcript (a target of miR165/6) to assay the slicer activity of AGO1-miR165/6 (a scheme of the procedure is shown in Fig 8A). While the AGO1 IP was able to cleave the *PHB* RNA, the AGO1 IP after the tailing reaction with URT1 failed to cleave the *PHB* RNA (Fig 8B). miR156/6 and AGO1 were present after the URT1 reaction, as shown by northern blotting and western blotting, respectively (Fig 8C and 8D). Tailing of miR165/6 by URT1 was also visible by northern blotting (Fig 8C). To confirm that the loss of cleavage activity was due to miR165/6 tailing, we conducted the tailing reaction with heat-inactivated URT1 or the URT1 catalytic mutant. Neither enzyme affected the cleavage activity of miR165/6 (Fig 8B–8D). This result was initially unexpected, as we did not expect 100% tailing of miR165/6 by URT1. But upon closer examination of the profiles of miR165/6 in northern blotting (Fig 8C), it seemed that most of the miR165/6 species were tailed by a small number of nucleotides, as the profiles looked different when functional URT1 was used.

## Discussion

### URT1 and HESO1 are nucleotidyl transferases with similar and distinct properties

In this study, we identify URT1 as a nucleotidyl transferase that tails unmethylated miRNAs and show that URT1 acts with the previously identified nucleotidyl transferase HESO1 in parallel or sequentially to tail various forms of the same miRNA *in vivo*. As nucleotidyl transferases, URT1 and HESO1 are similar in that both prefer UTP over the other three nucleotides and both are completely inhibited by 2’-*O*-methylation in their substrate RNA. These biochemical properties are consistent with molecular genetic observations that uridylated miRNAs are prevalent only in *hen1* mutants [8].

URT1 and HESO1 also exhibit distinct substrate preferences, especially with regard to the identity of the 3’ nucleotide in the substrate RNA. Our biochemical studies suggest that HESO1 has the strongest preference for RNA ending in U whereas URT1 has the strongest preference for RNA ending in A. This preference, together with the tendency to use UTP instead of other nucleotides for both enzymes, could explain the predominant role of HESO1 in miRNA tailing *in vivo*. Once HESO1 adds a U to the end of a miRNA, regardless of the nature of its 3’ nucleotide, the resulting tailed miRNA ends in a U and is likely a preferred substrate for HESO1. Therefore, HESO1 is likely able to hold on to the product of its reaction and use it as a substrate, i.e., HESO1 could be a processive enzyme. URT1, on the other hand, prefers A-ending miRNAs, but once it adds a U, the resulting miRNA is not a good substrate. In fact, this can be seen in the activity assay in Fig 5B (lanes 6, 11, 16), where the species with a few additional nucleotides persist. This suggests that URT1 is probably not a processive enzyme. Small RNA profiling in *hen1-8* and *hen1-8 urt1-1* in this study is consistent with this conclusion. We found that that URT1 mono-uridylates miR158, miR171a, and miR173 *in vivo*. After mono-uridylation of miR171a and miR173 by URT1, HESO1 further uridylates the mono-uridylated, U-ending miRNAs *in vivo*.

When both enzymes are active *in vivo*, one would expect HESO1 to out-compete URT1 in miRNA tailing. When one enzyme is knocked out, such as in *hen1-8 heso1-1* or *hen1-8 urt1-1*, the remaining enzyme should in theory have access to all miRNAs. In *hen1-8 heso1-1*, miRNA tailing is drastically reduced with monouridylation being the predominant forms left [10]. This is consistent with the conclusion that URT1 is not a highly processive enzyme. In *hen1-8 urt1-1*, most miRNAs are still fully tailed, suggesting that HESO1 can act on most miRNAs. The ten miRNAs that show reduced tailing in *hen1-8 urt1-1* must be refractory to HESO1 activity. In fact, most of the miRNAs end in C, A or G (S8 Fig), which are non-preferred 3’ nucleotides for HESO1. The exceptions are miR845a and b, for which the affected forms in *hen1-8 urt1-1* end in U (S6 Fig). It is unclear why HESO1 does not act effectively on these miRNAs *in vivo*.

It should be noted that the preferences for the 3’ ending nucleotide cannot fully account for the substrate preferences of the enzymes, nor their processivity. For example, for both URT1 and HESO1, miR158-1, which ends in C, is not as good a substrate as miR158A-C, in which the last nucleotide of miR158 is mutated to C (Fig 5). The sequence of the 3’ region of the miRNA substrate probably also matters.

There are ten potential nucleotidyl transferases in the genome. Two of them are now shown to tail unmethylated miRNAs. HESO1 and URT1 also act on mRNAs or mRNA fragments [16,22]. Both HESO1 and URT1 are completely inhibited by 2’-*O*-methylation in their miRNA substrates, it would be interesting to know whether any of the eight proteins can act on methylated miRNAs. It would also be interesting to know what RNA substrates these enzymes act on, such as siRNAs, other noncoding RNAs, or mRNAs.

### Tailing of AGO1-bound miRNAs and the fate of the tailed miRNAs

An important discovery of this study is that both HESO1 and URT1 can act on native miRISCs to tail miRNAs and that the tailed miRNAs remain bound by AGO1 *in vitro*. It is surprising that HESO1 is able to add more than 100 nucleotides to AGO1-bound miRNAs *in vitro*, as miRNAs in *hen1* mutants have much shorter tails (usually less than eight nucleotides [10,15]). As tailed miRNAs in *hen1* mutants are bound by AGO1 *in vivo* [12,15], it is likely that only miRNAs with short tails are found *in vivo* because only these species can be stably accommodated by AGO1, i.e., with the 5’ and 3’ ends of the miRNAs in the respective binding pockets in AGO1. miRNAs with long tails are unlikely to have their 3’ end protected by the PAZ domain of AGO1 and are likely susceptible to degradation or 3’ trimming to generate miRNAs with shorter tails.

Given that HESO1 and URT1 can act on AGO1-bound miRNAs and miRNAs with short tails can be accommodated by AGO1, degradation may not be the only outcome of miRNA tailing. In this study, we show that tailing of AGO1-bound miR165/6 by URT1 nearly abolishes its target RNA cleavage activity. Most of the AGO1-bound miR165/6 species had short tails after the URT1 reaction and were bound by AGO1, yet they lost their activity. This suggests that the tailed species are not properly positioned in AGO1 for optimal slicer activity. Our results on the expression of At3g03580, a target of miR158, are also consistent with tailed miR158 species not being functional (Fig 2E). We suspect that miRNA tailing leads to reduced miRNA activity in general, but an exception is miR171a. Mono-uridylation of 21-nt miR171a by URT1 in *hen1* mutants triggers the production of phasiRNAs from a miR171a target gene. This demonstrates that mono-uridylated miR171a is functional *in vivo* and that 3’ tailing alters the activities of this miRNA.

## Materials and Methods

### Plant materials

All *Arabidopsis* strains are in the Columbia background except for *hen1-2 heso1-2 urt1-3* (generated in the companion manuscript), which is in the Landsberg background. Seeds of nine *ntp* mutants were obtained from the Gabi-Kat collection or ARBC collection (see S1 Table for details). *hen1-8 ntp* double mutants were made by crossing *hen1-8* with *ntp* mutants and genotyping F2 populations for plants homozygous for both *hen1-8* and *ntp* mutations. Plants were grown under long day (16 h light/ 8 h darkness) conditions at 22°C.

### Plasmid construction

Towards the construction of the *pURT1*:*URT1*-GFP plasmid, a 1.4 kb fragment upstream of the *URT1* coding region was amplified by PCR with primers 3-Kpn1-1610F(PF) and URT1-pst1-PR as the promoter, and cloned into TSK108, a pENTRY-D-topo-based Gateway entry vector, at *Kpn*I and *Pst*I sites. Subsequently, the cDNA (without the stop codon) was amplified by PCR with primers URT1-pst1-CDSF and URT1-SPE1-CDSR and inserted next to the promoter at *Pst*I and *Spe*I sites. Then the promoter-cDNA fragment was moved into pMDC107 [24] by LR reaction. The plasmid was used to transform *hen1-8 urt1-1* plants by Agrobacterium (GV3101)-mediated floral dip transformation.

To construct the pET32a-URT1_WT plasmid for the expression of wild-type URT1 in *E*. *coli*, the coding sequence of *URT1* was amplified using primers URT1-sac1-F and URT1-Xho1-R and cloned into pET-32a. The orientation of the insertion was confirmed by sequencing.

To construct the pET32a-URT1_M plasmid for the expression of the catalytic mutant of URT1 in *E*. *coli*, mutagenesis of *URT1* was first performed by PCR with primers that incorporated the D491A and D493A mutations in the *URT1* coding sequence. Primers URT1-Sac1-F and URT1-DADA-R were used to generate the 5’ *URT1* fragment; primers URT1-DADA-F and URT1-Xho1-R were used to generate the 3’ *URT1* fragment. The two fragments were annealed and used as the template to amplify the full-length *URT1m* using URT1-Sac1-F and URT1-Xho1-R as primers. This full-length fragment was cloned into pET-32a. The orientation of the insertion was confirmed by sequencing. See S3 Table for sequences of primers.

### Quantitative RT-PCR analysis

Reverse transcription and real-time PCR were performed as described [25]. Total RNAs were prepared from inflorescences, and converted to cDNAs using Superscript III reverse transcriptase (Invitrogen) and oligo-dT. The cDNAs were then used as templates for real-time PCR with gene-specific primers. Real-time PCR was performed in triplicates using on a Biorad IQcycler apparatus with the Quantitech SYBR green kit (BioRad). The *ACTIN8* gene was used as the internal control. Primers used are listed in S3 Table.

### Northern blotting

RNA isolation and northern blotting to detect small RNAs were performed as described [26]. Total RNAs were extracted from inflorescences or AGO1 immunoprecipitate using the TRIzol reagent (Invitrogen). 5′-end-labeled (^32^P) antisense DNA oligonucleotides were used to detect miRNAs. Sequences of probes are shown in S3 Table.

### Phylogenetic analysis

A phylogenetic analysis was performed for ten putative *Arabidopsis* nucleotidyl transferases, MUT68 from *Chlamydomonas reinhardtii*, TUT4 from *Homo sapiens*, and Cid1 from *Schizosaccharomyces pombe*. Sequences corresponding to the NT_PAP_TUTase (cd05402) domain [27] from these proteins were aligned using ClustalW (http://www.ebi.ac.uk/Tools/msa/clustalw2/) with default parameters [28]. The phylogenetic tree was generated using MEGA5 [29].

### Small RNA library construction and sequencing

Cloning of small RNAs was carried out as described [30]. 50 μg total RNAs were resolved in a 15% polyacrylamide gel and 15–40 nt small RNAs were eluted from an excised gel piece. The small RNAs were ligated sequentially with the 3' and 5' adapters using the Small RNA Sample Preparation Kit (Illumina). Sufficient amounts of products were obtained by performing a reverse transcription reaction followed by a low-cycle PCR amplification. The libraries were barcoded and sequenced in one lane on an Illumina HiSeq2000.

### Analysis of miRNA 3’ truncation and 3’ tailing

Small RNA reads that passed Illumina’s quality control were separated into different genotypes according to the indexes. These high-quality small RNA reads were then mapped to the TAIR10 *Arabidopsis* genome using Bowtie [31]. The reads that matched to annotated tRNAs/rRNAs/snRNAs/snoRNAs were removed. The total numbers of reads that passed the quality and tRNA/rRNA filters for the various genotypes and replicates are listed in S2 Table.

To analyze miRNA 3’ tailing, a previously-developed bioinformatics pipeline was employed [15]. Briefly, any small RNA read that could not be perfectly mapped back to the genome was trimmed one nucleotide at a time from the 3’ end until the remaining sequence was perfectly mapped to the genome. The trimmed 3’ sequence was designated as the “tail” whereas the longest 5’ genome-mapped component (the “head”) was compared to all annotated miRNAs in miRBase to ascertain from which miRNAs they were derived.

To quantify the extent of tailing, *Arabidopsis* miRNAs annotated in miRBase v17 [32] were examined. Small RNA reads with the 5’ head perfectly aligning to each one of the annotated miRNAs were identified, and the amount of tailing was calculated as the ratio of the number of reads with tails to that of total reads of variants derived from a particular miRNA.

Small RNA phasing analysis was conducted as previously described [33]. Small RNA abundances from the antisense strand were combined with those of the sense strand, based on an anticipated 2 nt overhang at their 3’ ends, which is a typical feature of Dicer-produced small RNA duplexes. Phasing scores and combined abundances of small RNAs were graphed using a customized Perl script.

### Protein purification and enzymatic assays

The pET32a-URT1_WT and pET32a-URT1 _M plasmids were transformed into the *E*. *coli* strain BL21 Star™(DE3) for protein expression. The transgenic *E*. *coli* strains were cultured at 30°C until the OD reached 0.5. IPTG was added to a final concentration of 0.1 mM and the culture was incubated at 16°C overnight. The recombinant proteins were purified using Ni-NTA agarose (Invitrogen) under native conditions following the manufacturer's instructions. After the extract containing a recombinant protein was loaded onto the column, the column was washed four times with the following wash buffers: wash buffer 1 (200 mM NaCl, 50 mM Tris-HCl pH 8.0, 40 mM imidazole), wash buffer 2 (200 mM NaCl, 50 mM Tris-HCl pH 8.0, 60 mM imidazole), wash buffer 3 (200 mM NaCl, 50 mM Tris-HCl pH 8.0, 100 mM imidazole), and wash buffer 4 (200 mM NaCl, 50 mM Tris-HCl pH 8.0, 120 mM imidazole). Then the recombinant protein was eluted with elution buffer (200 mM NaCl, 50 mM Tris-HCl pH 8.0, 500 mM imidazole).

The URT1 enzymatic activity assays in Fig 3 were conducted in 10 μl reaction mixtures containing 4.8 pmole recombinant wild-type or mutant URT1, 1 μl of 40 mM miR173 and 1 mM of different nucleotide triphosphates in the URT1 reaction buffer (20 mM Tris-HCl pH 8.0, 50 mM NaCl, 0.7 mM MnCl2, 10 mM MgCl2, 0.5 mM DTT, and 100 μg/mL BSA). Reactions were conducted at room temperature for 0–20 min, and stopped by the addition of formamide dissolved in RNA loading dye. The reaction mixtures were denatured at 95°C for 5 min, incubated on ice for 5 min, and loaded on a 15% polyacrylamide urea gel. After gel electrophoresis, the gel was stained by ethidium bromide and imaged with a Gel Doc™ XRS+ imaging system (BIO-RAD).

The URT1 enzymatic activity assays in Fig 5 were conducted as described above except that the RNA oligonucleotide substrates were 5’ ^32^P-labeled and the products were visualized by autoradiography. 1 pmole of RNA oligonucleotides and 8 pmole of URT1 were present in the reactions. The HESO1 enzymatic assays in Fig 5 were performed as described [10]. 1 pmole of RNA oligonucleotides and 2 pmole of HESO1 were present in the reactions. For the 5’ labeling of RNA oligonucleotides, a 50 μl reaction mixture containing 100 μM oligonucleotide, 20 U T4 Polynucleotide Kinase (New England Biolabs), 1X T4 PNK buffer and 4 μl ATP [γ-^32^P] (3000Ci/mmol 10mCi/ml from PerkinElmer) was incubated at 37°C for 1 hour. The RNA was then purified with Illustra MicroSpin™ G-25 Columns (GE Healthcare) according to the manufacturer’s instructions. The RNA oligonucleotides were resolved in a 15% denaturing polyacrylamide gel, and gel pieces containing the full-length RNAs were excised, and the RNAs were eluted and used in the URT1 or HESO1 reactions.

The URT1 and HESO1 enzymatic activity assays in S6 Fig were conducted in 10 μl reaction mixtures containing 1 pmole of 5’ ^32^P-labeled miR158 or miR158-1, enzymes (see below), and 1 mM UTP in the URT1 reaction buffer (described above). For the reactions with miR158, 4 pmole URT1 alone, 4 pmole HESO1 alone, or 4 pmole URT1 and 4 pmole HESO1 together were used. The reaction mixtures were incubated for 1min at room temperature. For reactions with miR158-1, 8 pmole URT1 alone, 2 pmole HESO1 alone or 8 pmole URT1 and 2 pmole HESO1 together were used. The reaction mixtures were incubated at room temperature for 30 s.

### Immunoprecipitation of AGO1 and associated small RNAs

0.4–1.0g of seedling tissue from *hen1-2 heso1-2 urt1-3* plants was collected and ground to a fine powder in liquid nitrogen. 1 ml of IP buffer (50 mM Tris pH 7.5, 150 mM NaCl, 10% Glycerol, 0.1% NP-40, 4mM MgCl_2_, 5mM DTT and 1x protease inhibitor cocktail (Roche)) was added to the powder. The suspension was incubated at 4°C for 30 min and centrifuged at 16,000g for 15 min at 4°C. The supernatant was collected, filtered through 2 lays of Miracloth, and centrifuged as before. The supernatant was transferred into a new tube and pre-cleared with Dynabeads-Protein-A (Life Technologies) for 1 h at 4°C. The supernatant was separated from the beads using a magnetic stand and transferred into a new tube. The extract was then mixed with 2 μl of AGO1 antibody (Agrisera) and the mixture was incubated for 2 h with gentle shaking at 4°C. 20 μl pre-cleared Dynabeads-Protein-A was added and incubation was continued for another hour. The beads were washed four times with 1 ml wash IP buffer. Finally, the beads (i.e., AGO1 immunoprecipitate) were collected with a magnetic stand.

### Nucleotidyl transferase assays on AGO1-bound miRNAs

AGO1 IP was first performed as described above. The beads (AGO1 immunoprecipitate) were equally split into four portions, two for URT1 reactions and two for HESO1 reactions. The beads were washed three times with the URT1 or the HESO1 reaction buffer. After the washes, the beads were fully resuspended and equally split into two tubes (one for enzyme reaction and one for “no enzyme” control). Subsequently, the beads were collected and added to a 20 μl reaction mixture containing 0.7 pmole 6XHis-HESO1 or 38.5 pmole 6HisXURT1, reaction buffer (described above or in [10]) and cold UTP. The reactions were incubated for 40 min, the beads were separated from the supernatant with a magnetic stand, and RNA extraction was performed from the beads and the supernatant. Finally RNAs were resolved by gel electrophoresis and subjected to northern blotting to detect miR165/6 and miR172.

### Slicer assay on AGO1-bound miRNAs after URT1-mediated tailing

AGO1 IP was first performed as described above using 0.5g of *hen1-2 heso1-2 urt1-3* seedlings. The beads (AGO1 immunoprecipitate) were washed three times with the URT1 reaction buffer, and split into 3 equal portions, to be used for reactions with URT1, denatured URT1 (denaturing by boiling for 5 min), and the URT1 catalytic mutant (URT1m), respectively. Subsequently, each portion of beads was collected and added to a 60 μl reaction mixture containing 19 pmole 6XHis-URT1 (or denatured URT1 or URT1m), reaction buffer (described above) and cold UTP. The reactions were carried out at room temperature for 40 min; afterwards the beads were collected and washed 3 times with the IP buffer. The beads in each tube were further split into three equal portions, with one portion to be used for western blotting to quantify AGO1, another portion to be used for northern blotting to quantify AGO1-bound miR165/166, and the third portion to be used for the slicer assay. The beads in the third portion were collected and added to a 20 μl reaction mixture containing 15μl IP buffer, 4μl 5 X cleavage buffer and 1μl uniformly radiolabeled *PHB* (see the in vitro transcription section below), a target of miR165/6. The reactions were incubated for 1h, and RNA extraction was performed. Finally. RNAs were resolved by gel electrophoresis and visualized by autoradiography. 5 X cleavage buffer: 5 mM ATP, 1 mM GTP, 6 mM MgCl2, 125 mM creatine phosphate, 150 mg/mL creatine kinase and 2 unit/mL RNasin RNase Inhibitor.

### *In vitro* transcription

The template for *in vitro* transcription is generated by PCR using primers specific for the miR165/166 target gene *PHB*. The PCR-amplified *PHB* fragment is about 300 bp that contains the miR165/166 target region. T7 promoter sequence is added to the 5’end of the forward primer. The Riboprobe T7 kit (Promega) was used to generate transcripts from this PHB fragment. In a 20 μl reaction, 1μl DNA template and 3μl UTP [α-^32^P] (3000Ci/mmol 10mCi/ml from PerkinElmer) was added. The reaction was incubated at 37°C for 1 hour, and then stopped by the addition of formamide dissolved in RNA loading dye. The reaction mixtures were denatured at 95°C for 5 min, incubated on ice for 5 min, and loaded on a 5% polyacrylamide urea gel. The band of the expected size was excised and the RNAs were eluted and used in the cleavage assay.

### Accession numbers

The small RNA sequencing data in this study have been deposited at the GEO repository under the ID# GSE61362.

## Supporting Information

S1 Fig URT1 is a member of the nucleotidyl transferase gene family in *Arabidopsis*. (A) Phylogenetic relationships among ten known or potential nucleotidyl transferases from *Arabidopsis*. Three known nucleotidyl transferases from other organisms, TUT4, Cid1, and MUT68, were included in the phylogenetic analysis. All *Arabidopsis* proteins are indicated by gene name followed by gene ID. The amino acid sequences of the nucleotidyl transferase (NT) domain of the proteins were used for the analysis. Evolutionary distance is indicated by the scale bar. The numbers at the branches represent bootstrap values (the number of times of branch occurrence in 1000 replicates). (B) A diagram of the URT1 protein showing the NT domain (also known as PAP or TUTase domain). The sequence of a portion of the domain is shown. Two conserved aspartic acid residues (arrows) in the NT domain predicted to be part of a metal binding triad in the wild type (W) protein were mutated to alanine in the mutant protein (M). (C) Recombinant 6XHis-URT1 and 6XHis-URT1m (mutant version as in (B)) purified from *E*. *coli*. The recombinant proteins were resolved in an SDS-PAGE gel and stained with Coomassie Blue.
(JPG)

S2 Fig Effects of *heso1-1* and *urt1-1* on plant morphology. Four-week-old wild-type (Col), *hen1-8*, *hen1-8 heso1-1*, and *hen1-8 urt1-1* plants are shown.
(JPG)

S3 Fig Evaluation of effects of mutations in nine nucleotidyl transferase genes on miRNA tailing by small RNA sequencing. Among these genes, only *URT1* and *MEE44* were given gene names; the others are referred to as *NTP* genes here (see S1 Table for the gene IDs). Results on ten abundant miRNAs are diagramed here. In the diagrams, the X and Y axes represent the number of nucleotides truncated from, and tailed onto, the miRNA 3’ end, respectively. The sizes of the circles indicate the relative abundance of the miRNA variants. Note that the nine double mutants were processed in two separate experiments, each including the *hen1-8* control. The samples processed in the same experiment are shown together.
(JPG)

S4 Fig The status of miRNA 3’ truncation and tailing in various genotypes as determined by small RNA sequencing. In the diagrams, the X and Y axes represent the number of nucleotides truncated from, and tailed onto, the miRNA 3’ end, respectively. The sizes of the circles indicate the relative abundance of the miRNA variants. (A) The status of miR158a 3’ truncation and tailing is nearly identical in *hen1-8* and *hen1-8 mee44*. *MEE44* is one of the ten genes encoding potential nucleotidyl transferases. (B) The status of miR158a 3’ truncation and tailing in wild type (Col) and *urt1-1*. Please refer to the publication by Zhai et al. (Plant Cell 25, 2417–2428) for the status of miR158a in *heso1-1* as a comparison to the results on *urt1-1*. (C-D) The status of miR173 (C) and miR171a (D) 3’ truncation and tailing in wild type (Col) and *urt1-1*.
(TIF)

S5 Fig Levels of miR173 in various genotypes as determined by northern blotting. (A) The increased levels of miR173 in *hen1-8 urt1-1* were rescued by the *pURT1*:*URT1-GFP* transgene. *pURT1*:*URT1-GFP1* and *pURT1*:*URT1-GFP2* are two independent T1 lines of *hen1-8 urt1-1* containing the transgene. (B) The *heso1-1* or *urt1-1* mutation does not affect the levels of miR173 in the wild-type *HEN1* background. The numbers below the miR173 image represent mean+/-SD; the SD was calculated from three biological replicates.
(JPG)

S6 Fig URT1 and HESO1 work coordinately to tail the miRNA. 5’ ^32^P-labeled miR158 or miR158-1 was incubated with buffer alone (lane 1), URT1 alone (lane 2), URT1 and HESO1 (lane 3), or HESO1 alone (lane 4). See Methods for the details of the reactions.
(JPG)

S7 Fig AGO1 immunoprecipitation (IP). AGO1 was immunoprecipitated from *hen1-2 heso1-2 urt1-3* seedlings. The input and IP products were subjected to western blotting with anti-AGO1 and anti-HSC70 antibodies. The IP was used for tailing reactions with URT1 or HESO1 in Fig 6.
(JPG)

S8 Fig Sequences of miRNAs that show reduced 3’ tailing in *hen1-8 urt1-1* relative to *hen1-8*. As tailing occurs on both full-length and 3’ truncated forms in *hen1-8* and not all forms of a specific miRNA are affected by the *urt1-1* mutation, the forms affected are indicated by their 3’ end nucleotides in red color.
(JPG)

S1 Table Information on *NUCLEOTIDYL TRANSFERASE PROTEIN* (*NTP*) genes and mutants (PDF)

S2 Table The processing of reads from small RNA high throughput sequencing (PDF)

S3 Table Sequences of DNA oligonucleotides used in this study (PDF)
