# Supplementary figures and images for: Distinct and Cooperative Activities of HESO1 and URT1 Nucleotidyl Transferases in MicroRNA Turnover in Arabidopsis
Source: PLoS Genet. 2015 Apr 30;11(4):e1005119. doi: 10.1371/journal.pgen.1005119 (PMC4415760; doi:10.1371/journal.pgen.1005119)

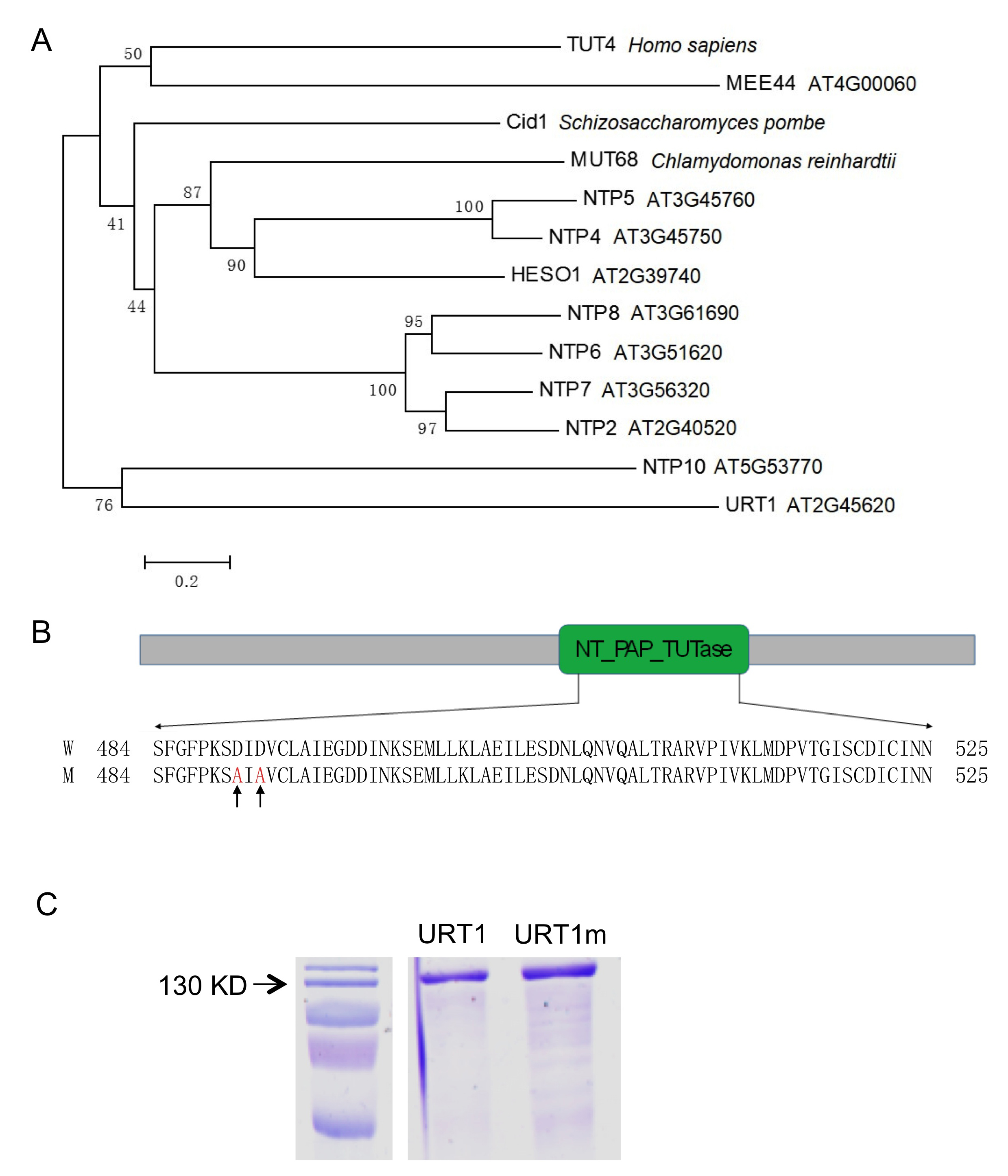

Supplement: S1 Fig — (A) Phylogenetic relationships among ten known or potential nucleotidyl transferases from Arabidopsis. Three known nucleotidyl transferases from other organisms, TUT4, Cid1, and MUT68, were included in the phylogenetic analysis. All Arabidopsis proteins are indicated by gene name followed by gene ID. The amino acid sequences of the nucleotidyl transferase (NT) domain of the proteins were used for the analysis. Evolutionary distance is indicated by the scale bar. The numbers at the branches represent bootstrap values (the number of times of branch occurrence in 1000 replicates). (B) A diagram of the URT1 protein showing the NT domain (also known as PAP or TUTase domain). The sequence of a portion of the domain is shown. Two conserved aspartic acid residues (arrows) in the NT domain predicted to be part of a metal binding triad in the wild type (W) protein were mutated to alanine in the mutant protein (M). (C) Recombinant 6XHis-URT1 and 6XHis-URT1m (mutant version as in (B)) purified from E. coli. The recombinant proteins were resolved in an SDS-PAGE gel and stained with Coomassie Blue. (JPG) [file pgen.1005119.s001.jpg]

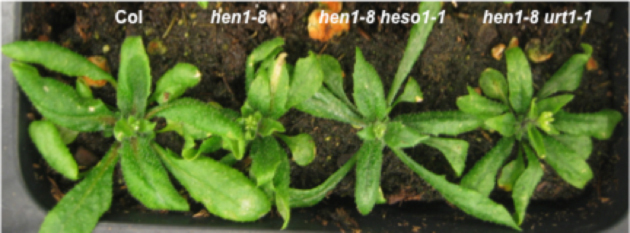

Supplement: S2 Fig — Four-week-old wild-type (Col), hen1-8, hen1-8 heso1-1, and hen1-8 urt1-1 plants are shown. (JPG) [file pgen.1005119.s002.jpg]

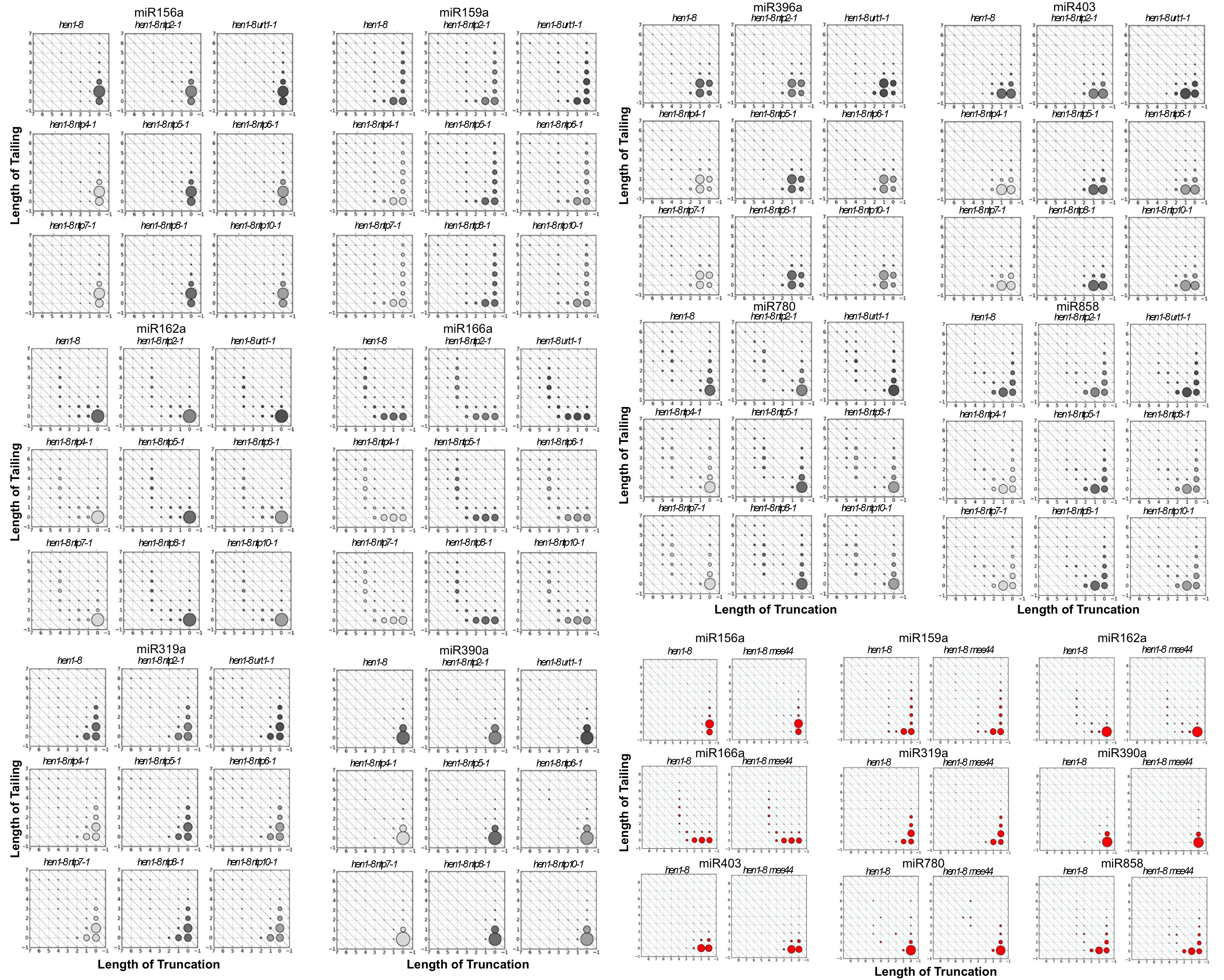

Supplement: S3 Fig — Among these genes, only URT1 and MEE44 were given gene names; the others are referred to as NTP genes here (see S1 Table for the gene IDs). Results on ten abundant miRNAs are diagramed here. In the diagrams, the X and Y axes represent the number of nucleotides truncated from, and tailed onto, the miRNA 3’ end, respectively. The sizes of the circles indicate the relative abundance of the miRNA variants. Note that the nine double mutants were processed in two separate experiments, each including the hen1-8 control. The samples processed in the same experiment are shown together. (JPG) [file pgen.1005119.s003.jpg]

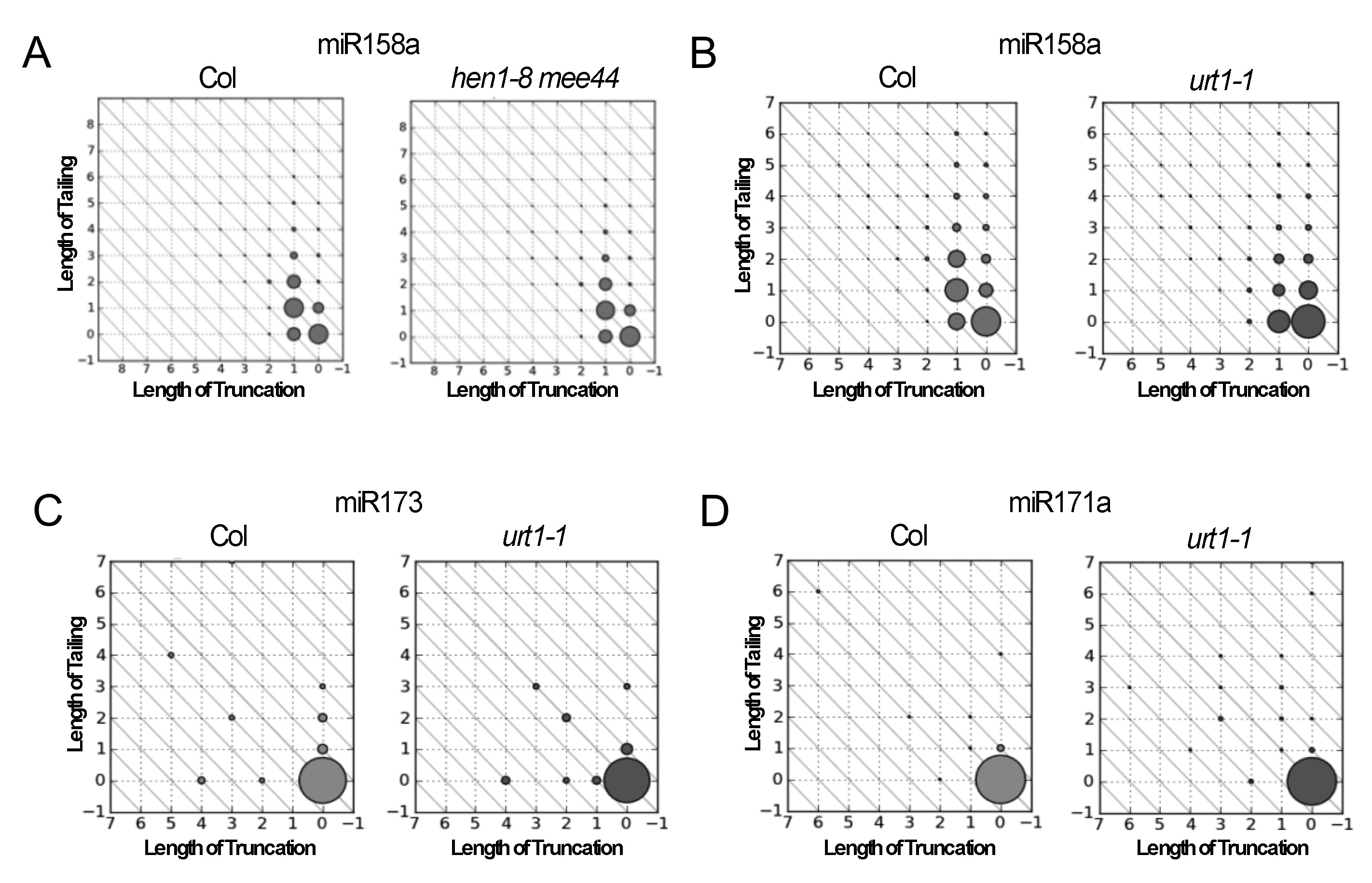

Supplement: S4 Fig — In the diagrams, the X and Y axes represent the number of nucleotides truncated from, and tailed onto, the miRNA 3’ end, respectively. The sizes of the circles indicate the relative abundance of the miRNA variants. (A) The status of miR158a 3’ truncation and tailing is nearly identical in hen1-8 and hen1-8 mee44. MEE44 is one of the ten genes encoding potential nucleotidyl transferases. (B) The status of miR158a 3’ truncation and tailing in wild type (Col) and urt1-1. Please refer to the publication by Zhai et al. (Plant Cell 25, 2417–2428) for the status of miR158a in heso1-1 as a comparison to the results on urt1-1. (C-D) The status of miR173 (C) and miR171a (D) 3’ truncation and tailing in wild type (Col) and urt1-1. (TIF) [file pgen.1005119.s004.tif]

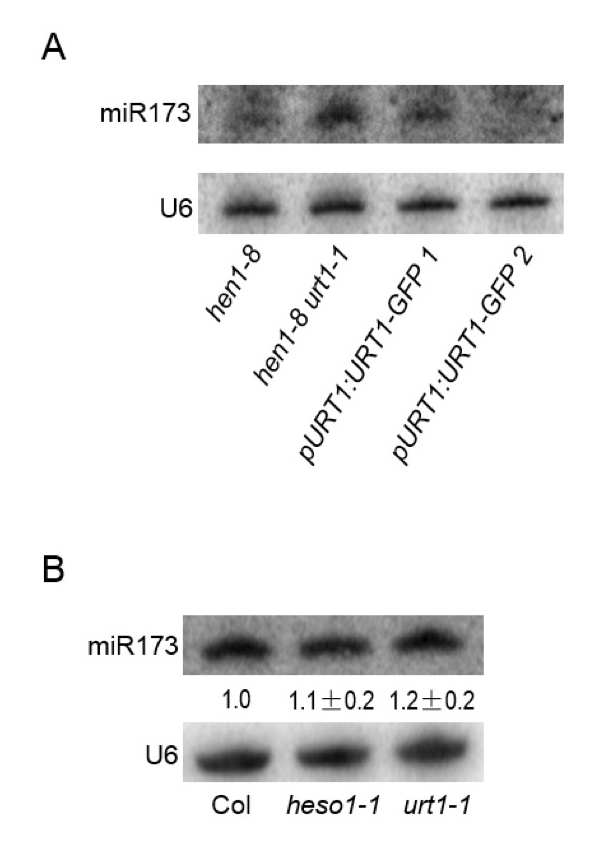

Supplement: S5 Fig — (A) The increased levels of miR173 in hen1-8 urt1-1 were rescued by the pURT1:URT1-GFP transgene. pURT1:URT1-GFP1 and pURT1:URT1-GFP2 are two independent T1 lines of hen1-8 urt1-1 containing the transgene. (B) The heso1-1 or urt1-1 mutation does not affect the levels of miR173 in the wild-type HEN1 background. The numbers below the miR173 image represent mean+/-SD; the SD was calculated from three biological replicates. (JPG) [file pgen.1005119.s005.jpg]

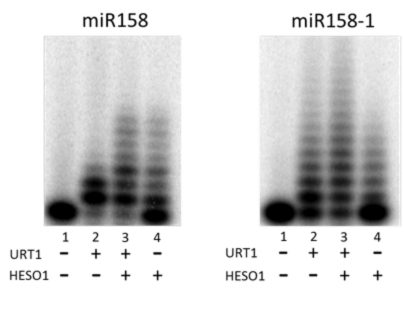

Supplement: S6 Fig — 5’ 32P-labeled miR158 or miR158-1 was incubated with buffer alone (lane 1), URT1 alone (lane 2), URT1 and HESO1 (lane 3), or HESO1 alone (lane 4). See Methods for the details of the reactions. (JPG) [file pgen.1005119.s006.jpg]

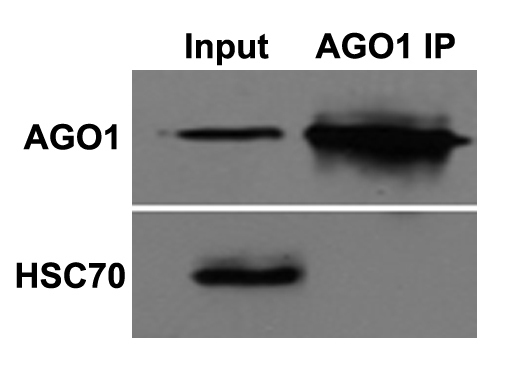

Supplement: S7 Fig — AGO1 was immunoprecipitated from hen1-2 heso1-2 urt1-3 seedlings. The input and IP products were subjected to western blotting with anti-AGO1 and anti-HSC70 antibodies. The IP was used for tailing reactions with URT1 or HESO1 in Fig 6. (JPG) [file pgen.1005119.s007.jpg]

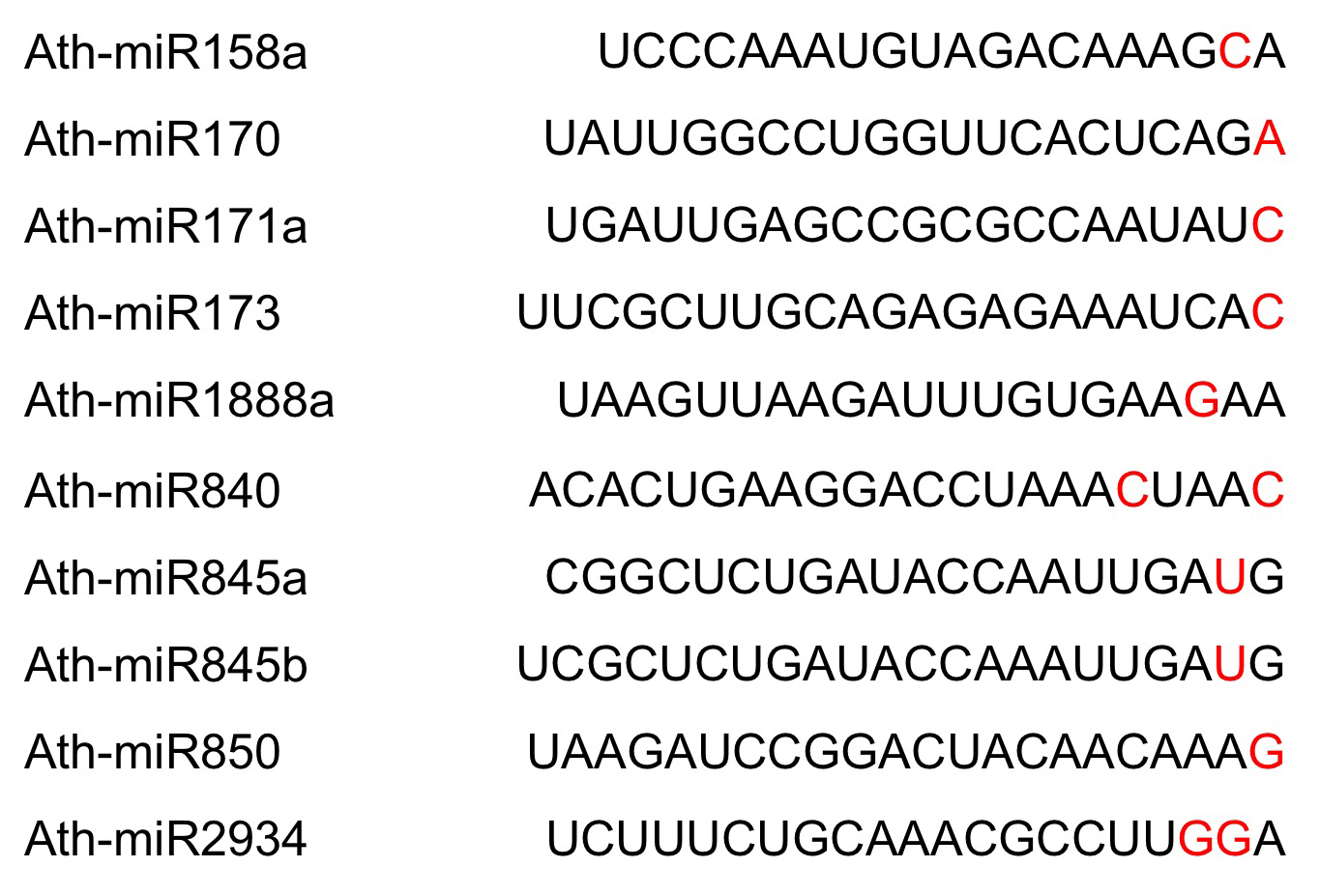

Supplement: S8 Fig — As tailing occurs on both full-length and 3’ truncated forms in hen1-8 and not all forms of a specific miRNA are affected by the urt1-1 mutation, the forms affected are indicated by their 3’ end nucleotides in red color. (JPG) [file pgen.1005119.s008.jpg]
