# Supplementary material for: Distinct and Cooperative Activities of HESO1 and URT1 Nucleotidyl Transferases in MicroRNA Turnover in Arabidopsis
Source: PLoS Genet. 2015 Apr 30;11(4):e1005119. doi: 10.1371/journal.pgen.1005119 (PMC4415760; doi:10.1371/journal.pgen.1005119)
Supplement: S1 Table — (PDF) [file pgen.1005119.s009.pdf]

Table S1. Information on *NUCLEOTIDYL TRANSFERASE PROTEIN* (*NTP*) genes and mutants

Gene name	Gene ID	Mutant stock number
HESO1	At2g39740	GK367H02
NTP2	At2g40520	CS810244
URT1	At2g45620	SALK_087647
NTP4	At3g45750	SALK_105213
NTP5	At3g45760	SALK_018808
NTP6	At3g51620	SALK_085337
NTP7	At3g56320	SALK_111188
NTP8	At3g61690	SALK_062369
MEE44	At4g00060	SALK_002103
NTP10	At5g53770	GK297E11
