# Supplementary material for: Distinct and Cooperative Activities of HESO1 and URT1 Nucleotidyl Transferases in MicroRNA Turnover in Arabidopsis
Source: PLoS Genet. 2015 Apr 30;11(4):e1005119. doi: 10.1371/journal.pgen.1005119 (PMC4415760; doi:10.1371/journal.pgen.1005119)
Supplement: S2 Table — (PDF) [file pgen.1005119.s010.pdf]

Table S2. The processing of reads from small RNA high throughput sequencing

Sample	Total Sequences	t/r/sn/snoRNA Matched Reads	Genome-matched Reads[&]	Distinct Genome-matched Reads
*hen1-8**	13,802,468	1,619,883	5,416,498	2,055,117
hen1-8 ntp2-1	15,247,683	1,592,099	6,195,419	2,213,528
hen1-8 urt1-1	14,085,835	1,368,955	6,025,554	2,132,191
hen1-8 ntp4-1	11,390,018	1,326,889	4,579,492	1,806,880
hen1-8 ntp5-1	15,088,782	2,407,093	5,625,995	2,037,369
hen1-8 ntp6-1	20,301,474	8,830,263	4,363,182	715,219
hen1-8 ntp7-1	10,979,136	1,349,399	4,370,837	1,777,325
hen1-8 ntp8-1	20,925,152	1,892,603	8,668,774	2,860,100
hen1-8 ntp10-1	16,235,160	1,840,714	6,333,711	2,321,432
*hen1-8**	6,693,761	1,121,306	2,965,426	1,244,773
hen1-8 heso1-1	10,860,015	1,656,142	5,652,208	1,706,609
hen1-8 mee44	8,855,679	1,536,828	3,536,867	1,486,276

* The control *hen1-8* was included in each of two small RNA library construction experiments. Each *hen1-8* sample was processed at the same time as, and serves as the control for, the genotypes below.

[&] This column represents non-t/r/sn/snoRNA, genome-matched reads.
