# Supplementary material for: Distinct and Cooperative Activities of HESO1 and URT1 Nucleotidyl Transferases in MicroRNA Turnover in Arabidopsis
Source: PLoS Genet. 2015 Apr 30;11(4):e1005119. doi: 10.1371/journal.pgen.1005119 (PMC4415760; doi:10.1371/journal.pgen.1005119)
Supplement: S3 Table — (PDF) [file pgen.1005119.s011.pdf]

Table S3. Sequences of DNA oligonucleotides used in this study

Primer name	Oligonucleotide sequence (5’-3’)	Usage
GK367_GT-F	CTGGTTCTGTGATTGTTAGGTG	Genotyping
GK367_GT-R	GAGACCAACAGCTCCGAGA	Genotyping
Gabi-kat_T-DNA	ATATTGACCATCATACTCATTGC	Genotyping
LBa1	TGGTTCACGTAGTAAACCATCG	Genotyping
S087647LP	ACCTGTGGATGTCATTTCGAC	Genotyping
S087647RP	GTTTCCTCAATTTCCGCCTAG	Genotyping
T13K14-P19	TAGTCTCATCAAGTTATGTCT	Genotyping
T13K14-P20	GCAGAGAAGCGTGTTCAATC	Genotyping
NF-105213	ATCAGGAATCTCTGGGTAGAC	Genotyping
NR-105213	TCTGTTTGCGATGTGTTCCAG	Genotyping
CS810224F	GCTTTTGGTAGAGACCATCTG	Genotyping
CS810224R	GTCACACTACGTCCAAGGTTG	Genotyping
S111188LP	TGGACAACTGGTCTAAAACCG	Genotyping
S111188RP	AAGCCTACTGCACAATTGTCC	Genotyping
GTAT5G5370F	GATTGCTAAGGCTCGTGTACCA	Genotyping
GTAT5G5370R	GTTATGCTCAGGTGCGCTTCGA	Genotyping
S074440LP	CACTTCCCCAGGTAATTTTGG	Genotyping
S074440RP	TTAGTTTTGGCATTTTCTCCG	Genotyping
S018808FP-AS	CTGGTTAAACCAATGTGAGGA	Genotyping
S018808RP-P	ATCAAGAATCTCTTGGTAGAC	Genotyping
S085337LP	CACCCTGGAAATGAAGAAATG	Genotyping
S085337RP	CCTCGTGGCTTAGGTAATTCC	Genotyping
3-Kpn1-1610F(PF)	CGGGGTACCCAAAGACGTAACCATTA AC	Plasmid construction
URT1-pst1-PR	AACTGCAGGGAGGAGGAGAGAAAGTG AAGAT	Plasmid construction
URT1-pst1-CDSF	AACTGCAGATGGCGGACGGTGGGGCT GAACCT	Plasmid construction
URT1-SPE1-CDSR	CTGGACTAGTGTTGTGGCCTTGTCCAT TATTATC	Plasmid construction
URT1-sac1-F	CATGAGCTCATGGCGGACGGTGGGGCT GAACCTCCAGCTC	Plasmid construction
URT1-xho1-R	CATCTCGAGCTAGTTGTGGCCTTGTCC ATTATTATC	Plasmid construction
URT1-DADA-F*	GGTTTCCCGAAGAGCgCcATCgCcGTTT GCCTTGCAATC	Plasmid construction
URT1-DADA-R*	GATTGCAAGGCAAACgGcGATgGcGCTC TTCGGGAAACC	Plasmid construction
PEG301 SEQ	GCATATCTCATTAAAGCAGG	Sequencing
PMDC107 SEQ	TCCCTTAAGCTCGATCCTGTT	Sequencing
SNTP2PRIMER1(C1 540)-R	CTGCCAGCTTTAACAACATCTCA	Sequencing
miRNA166_AS	GGGGAATGAAGCCTGGTCCGA	Northern blot
miRNA173_AS	GTGATTTCTCTCTGTAAGCGA	Northern blot
miRNA171a_AS	GATATTGGCGCGGCTCAATCA	Northern blot
miRNA158a_AS	TGCTTTGTCTACATTTGGGA	Northern blot

miRNA172_AS	ATGCAGCATCATCAAGATTCT	Northern blot
ASRP1511 (TAS2)	AAGTATCATCATTCGCTTGGA	Northern blot
AT3G03580RT-F	TATAGCTGGATCGAAGTCGG	Realtime PCR
AT3G03580RT-R	ACGCAATAGCAAGTCTCTCG	Realtime PCR
AT2G45620RT1F	TGCCAATGATGGGAAGAA	RT-PCR
AT2G45620RT1R	TAGCACAGGACCCGTAAA	RT-PCR
AT2G45620RT2F	TCTTCAACTACTGGGCTTAC	RT-PCR
AT2G45620RT2R	CTCCTCCCTCAACACTCT	RT-PCR
ACTIN 8 F	CACATGCTATCCTCCGTCTC	RT-PCR
ACTIN 8 R	CAATGCCTGGACCTGCTT	RT-PCR
PHB-T7-F	GGCACGTAATACGACTCACTATAGGGG TCTGTGGTCGTGAGTGGTC	PCR followed by in vitro transcription
PHB-R	GCCAAGATGGACGATCTTTG	PCR followed by in vitro transcription

* The lower case letters in the sequences represent mutated nucleotides.
